# Dietary Methionine Improves the European Seabass (*Dicentrarchus labrax*) Immune Status, Inflammatory Response, and Disease Resistance

**DOI:** 10.3389/fimmu.2018.02672

**Published:** 2018-11-20

**Authors:** Marina Machado, Rita Azeredo, Filipa Fontinha, Sergio Fernández-Boo, Luis E. C. Conceição, Jorge Dias, Benjamín Costas

**Affiliations:** ^1^Centro Interdisciplinar de Investigação Marinha e Ambiental, Matosinhos, Portugal; ^2^Instituto de Investigação e Inovação em Saúde, Universidade do Porto, Porto, Portugal; ^3^Instituto de Ciências Biomédicas Abel Salazar, Universidade do Porto, Porto, Portugal; ^4^Instituto de Biologia Molecular e Celular, Universidade do Porto, Porto, Portugal; ^5^Sparos Lda, Olhão, Portugal

**Keywords:** amino acids, cell proliferation, inflammation, immunostimulation, fish, nutraceutics

## Abstract

Methionine presents a pivotal role in the regulation of many cellular events with crucial impact on the immune system, such as in processes involved in the control of inflammation and polyamines synthesis. Accordingly, the present study aimed to assess the modulatory effects of dietary methionine on the European seabass (*Dicentrarchus labrax*) immune status, inflammatory response and disease resistance to *Photobacterium damselae* subsp. piscicida (*Phdp*). For this purpose, fish were randomly distributed in three independent groups (three replicates per group) and each was fed the corresponding diet: a control diet (CTRL) formulated to meet the established amino acid requirements for the species; a diet supplemented with methionine at 0.5% of feed weight relative to the CTRL diet (8.2% of methionine concentration above CTRL); and one supplemented with methionine at 1% of feed weight to the CTRL diet (11.8% of methionine concentration above CTRL). To evaluate the immune status of fish fed with each of the diets before being submitted to bacterial infection fish were sampled from each group at 2 and 4 weeks after the beginning of feeding. Non-sampled fish were injected intraperitoneally with *Phdp* (5 × 10^3^ cfu/fish) at 4 weeks after initiation of feeding and the inflammatory response (at 4, 24, and 48 h post-infection) and survival (lasting 21 days post-infection) evaluated. Fish hematological profile, peripheral cell dynamics, plasma humoral immune parameters, leucocyte migration to the inflammatory focus and head-kidney gene expression were evaluated. Results show that methionine dietary supplementation improves seabass cellular immune status without evidence of activation of pro-inflammatory mechanisms. Additionally, the observed enhanced immune status provided by methionine supplementation translated into an improved immune response to infection, as higher cellular differentiation/proliferation and recruitment to the inflammatory focus, improved plasma humoral immune parameters and modulation of key immune-related genes was observed. Lastly, after a bacterial challenge, higher survival was observed in fish fed supplemented diets, ultimately corroborating the positive effect of methionine administration for 4 weeks in the cellular immune status.

## Introduction

A dependency of the immune system upon the availability of amino acids (AA) has been associated to their role as signaling molecules essential for cellular function as reviewed in (1–4), but also as methyl group donors and precursors of physiological important molecules, such as hormones, bioactive amines, enzymes, neurotransmitters and nitric oxide. Several studies have reported that AA deficiency reduces their plasma concentration, ultimately compromising the immune system repertoire (5, 6). In fact, AA requirements may increase as a direct consequence of metabolic changes associated with inflammation and infection (7). Methionine is an example of an indispensable AA with a recognized role in the immune system and its dietary supplementation proved to enhance mammalian host immunity (8). By generating S-adenosylmethionine (SAM), methionine is a methyl group donor that participates in the methylation of DNA, ultimately influencing gene expression (8). Additionally, methionine takes part on the polyamine (i.e., spermidine and spermine) biosynthesis through the aminopropylation pathway, where decarboxylated SAM successively adds aminopropane to the forming polyamines, required for cell proliferation (9). During the transsulfuration pathway, methionine is also precursor of cysteine, one of the three glutathione (GSH) elements, a molecule involved in scavenging free radicals, hence protecting cells from oxidative stress during inflammation (1). Methionine also plays a pivotal role in processes responsible for the control of inflammation and apoptosis, such as protein ubiquitination and autophagy (10). By inducing SAM-mediated methylation, methionine has been shown to inhibit autophagy and promote growth in yeast (11). In fact, methionine and its downstream metabolite SAM are responsible for autophagy modulation (12). As a result, and knowing that the ideal inflammatory response is rapid, yet specific and self-limiting (13), methionine presents an important potential as immunomodulator during infection. Still, further in-depth studies are needed to understand the immune mechanisms that this particular AA is activating before and after infection episodes.

Methionine dietary immunomodulation also adds a practical perspective to modern animal production. For instance, the importance of methionine as a nutraceutical supplement to control enteric processes and oxidative stress in mammals has been recently reviewed (14). Moreover, Bunchasak (15) and Jankowski et al. (16) reviewed the many beneficial effects of dietary methionine (and other sulfur-containig AA) on poultry immune mechanisms and its use on poultry industry.

However, the role of methionine as an immunomodulatory additive in aquafeeds still needs to be explored so to improve sustainability and fish welfare in fish farming. Recent studies showed that methionine supplementation increase European seabass (*Dicentrarchus labrax*) cellular immune status as well as immune response to an inflammatory insult with UV-inactivated *Photobacterium damselae* subsp. *piscicida* (*Phdp*) (17). An increased peripheral leucocytes concentration was also observed in juvenile Jian carp (*Cyprinus carpio* var. Jian) after being fed graded levels of methionine hydroxy analog, a synthetic methionine source, resulting in increased survival rate and stronger humoral and cellular response after injection with *Aeromonas hydrophila* (18). Likewise, Tang and co-workers (19) observed an increase in plasma lysozyme activity, complement factors and IgM of Jian carp given dietary methionine supplementation during 8 weeks. Therefore, dietary methionine also seems to be an important nutritional additive for fish health management. The main goal of the present study was to gather evidence on the specific role of methionine orchestrating the European seabass immune response before and after a *Phdp* infection.

## Materials and methods

### Experimental design

**Figure 1:**
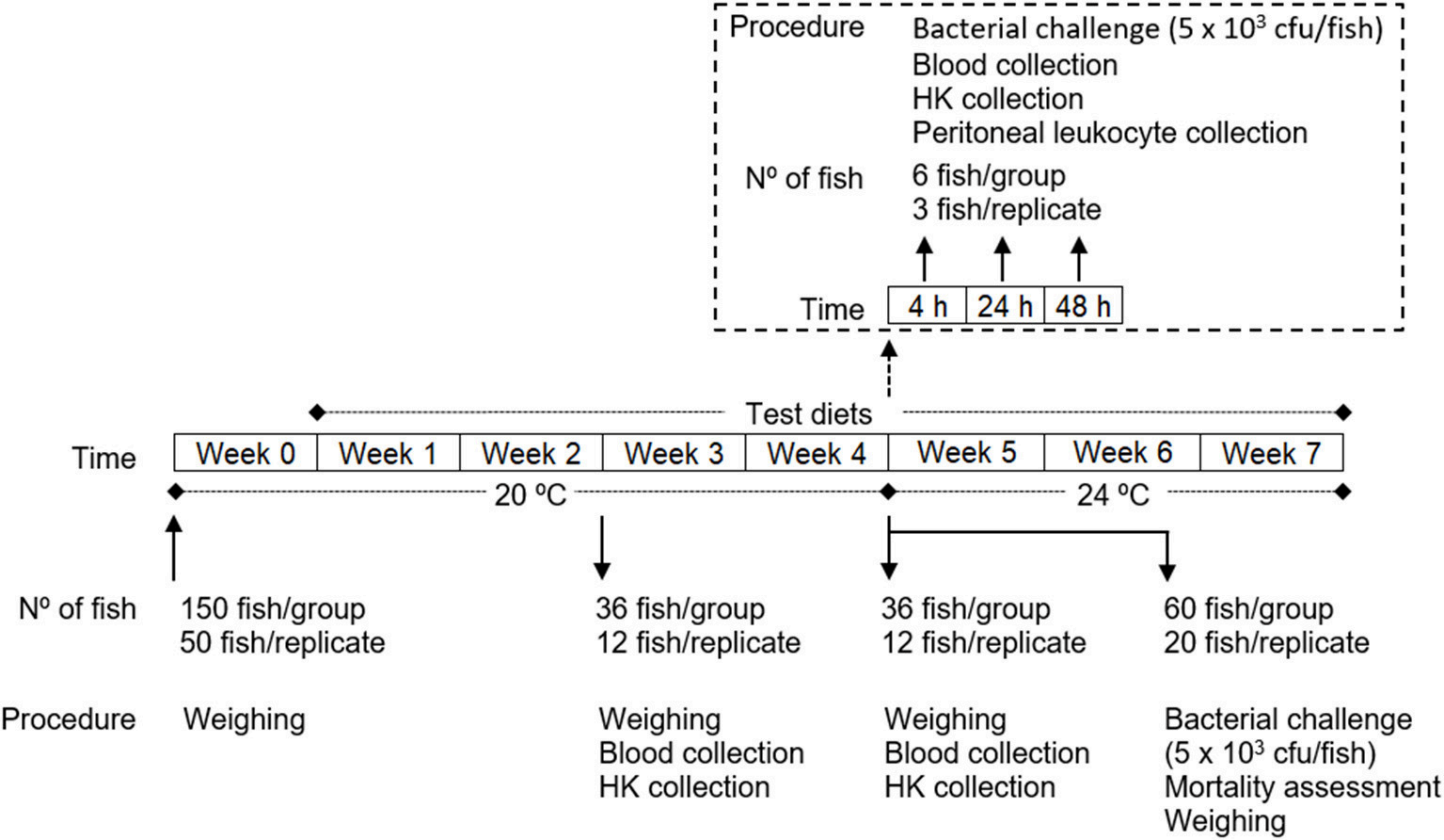
Experimental design.

European seabass juveniles were acquired to a certificated hatchery (MARESA, Spain) and maintained in quarantine for 2 weeks at the Instituto de Investigação e Inovação em Saúde (i3S; University of Porto, Portugal) fish holding facilities under the culture conditions described below. After this period, fish were weighed (Table 4) and randomly distributed into 9 tanks (200 l; 3 groups with 3 replicates of 50 fish each) of a recirculation seawater system in which O_2_ saturation (7.38 ± 0.01 mg/l), salinity (35 ppt) and photoperiod (10 h dark: 14 h light) were kept unchanged throughout the experiment (Figure 1). The temperature was maintained at 20 ± 0.5°C until the time the bacterial infection was carried out (4 weeks after feeding with the test diets), where it was increased to 24 ± 0.5°C until de end of the experiment so as to mimic the temperature increase which typically triggers piscine outbreaks. Ammonium and nitrite levels were kept below 0.025 and 0.3 mg l^−1^, respectively.

After 1 week, during which fish were all fed with the commercial diet with which they were being fed previously, the experiment was started by feeding of each group with the respective feed 3 times a day at an average ration of 2.5% biomass per day (daily adjusted ± 0.5% based on the assessment of the non-consumed feed): (i) one group was fed a control diet (CTRL); (ii) another group was fed a diet supplemented with 0.5% methionine of feed weight to the CTRL diet (MET0.5); and finally, (iii) another group was fed a diet supplemented with 1% methionine of feed weight to the CTRL diet (MET1).

At 2 and 4 weeks after feeding the test diets, 36 fish from each group (12 per replicate) were euthanized by an overdose of anesthetic (2-phenoxyethanol; Merck, ref. 807291, Germany), weighed, and collected blood and head kidney samples. Also at 4 weeks, fish that were not sampled (78 per group, 26 per replicate) were infected intraperitoneally (i.p.) with 100 μl of a *Phdp* suspension (5 × 10^4^ cfu ml^−1^). Of these, 60 fish per group (20 per replicate) were placed back in their tanks, feed replenished according to the previous regimen and mortality recorded for 3 weeks and the relative percentage survival (RPS) calculated. After euthanasia of the moribund fish, the animals were weighed and the presence of *Phdp* in the head-kidney checked by growing on TSA-2 plates. The remaining infected fish (6 per group, 3 per replicate) were re-allocated in a similar recirculation system (Temperature: 24 ± 0.5°C; Salinity: 35 ppt; Photoperiod: 10 h dark: 14 h light) according to dietary treatment and 6 fish per group were euthanized at 4, 24, and 48 h post-infection (time-course) and blood, head-kidney and peritoneal exudates sampled from each fish, to investigate the immunomodulatory effect of the diets during the initial inflammatory response to *Phdp*.

The experiments were approved by the i3S Animal Welfare Committee and carried out in a registered installation (license number 0421/000/000/2018). Experiments were performed by trained scientists in full compliance with national rules and following the European Directive 2010/63/EU of the European Parliament and the European Union Council on the protection of animals used for scientific purposes.

### Experimental diets

The 3 diets (Table 1) were formulated and manufactured by Sparos Lda (Olhão, Portugal). The CTRL diet was formulated to include an indispensable AA profile meeting the ideal pattern estimated for European seabass (20). According to results from previsous works (17, 21) two other diets were formulated (MET 0.5 and MET 1, respectively) to be identical to the CTRL but supplemented with DL-Methionine at 0.5 or 1% of feed weight, at the expenses of wheat gluten and wheat meal. After AA analysis the percentage of methionine in relation to the total AA amount was of 2.6% for CTRL and 2.8 and 3.2% for MET 0.5 and MET 1, respectively, presenting these diets 8.2 and 11.8% more methionine than CTRL.

**Table 1:** Ingredient and chemical composition of the experimental diets.

**Ingredients**	**CTRL**	**MET 0.5**	**MET 1**
		**%**
Fishmeal LT70 (South American)^1^	11.00	11.00	11.00
Fishmeal 60^2^	17.00	17.00	17.00
Soy protein concentrate^3^	12.00	12.00	12.00
Wheat gluten^4^	8.00	7.70	7.40
Corn glúten^5^	4.00	4.00	4.00
Soybean meal 48^6^	14.00	14.00	14.00
Rapeseed meal^7^	6.00	6.00	6.00
Wheat meal^8^	10.00	9.80	9.60
Fish oil^9^	8.50	8.50	8.50
Rapeseed oil^10^	5.00	5.00	5.00
Vitamin and mineral premix^11^	1.00	1.00	1.00
Brewer's yeast^12^	3.00	3.00	3.00
Soy lecithin^13^	0.50	0.50	0.50
DL-Methionine^14^	−	0.50	1.00
Total	100	100	100
Pellet size, mm	1.50	1.50	1.50
**PROXIMATE ANALYSES (% DRY WEIGHT)**			
Dry matter (g/100 g)	5.20	5.54	5.09
Protein (g/100 g)	45.83	45.62	46.25
Fat (g/100 g)	18.80	19.00	18.10
Ash (g/100 g)	7.74	7.58	7.81
Energy (kJ/g)	22.48	22.70	22.55

1 LT70 steam dried, 70.7% crude protein (CP), 8.1% crude fat (CF), Pesquera Diamante, Peru.

2. *COFACO 60: 62.3% CP, 8.4% CF, COFACO, Portugal*.

3. *Soycomil P: 63% CP, 0.8% CF, ADM, The Netherlands*.

4. *VITAL: 83.7% CP, 1.6% CF, ROQUETTE Frères, France*.

5. *Corn gluten meal: 61% CP, 6% CF, COPAM, Portugal*.

6. *Dehulled solvent extracted soybean meal: 47% CP, 2.6% CF, CARGILL, Spain*.

7. *Defatted rapeseed meal: 34% CP, 2% CF, Premix Lda, Portugal*.

8. *Wheat meal: 10.2% CP; 1.2% CF, Casa Lanchinha, Portugal*.

9. *SAVINOR UTS, Portugal*.

10. *Henry Lamotte Oils GmbH, Germany*.

11. *20 PREMIX Lda, Portugal: Vitamins (IU or mg/kg diet): DL-alpha tocopherol acetate, 100 mg; sodium menadione bisulphate, 25 mg; retinyl acetate, 20,000 IU; DL-cholecalciferol, 2,000 IU; thiamin, 30 mg; riboflavin, 30 mg; pyridoxine, 20 mg; cyanocobalamin, 0.1 mg; nicotinic acid, 200 mg; folic acid, 15 mg; ascorbic acid, 500 mg; inositol, 500 mg; biotin, 3 mg; calcium panthotenate, 100 mg; choline chloride, 1,000 mg, betaine, 500 mg. Minerals (g or mg/kg diet): copper sulfate, 9 mg; ferric sulfate, 6 mg; potassium iodide, 0.5 mg; manganese oxide, 9.6 mg; sodium selenite, 0.01 mg; zinc sulfate,7.5 mg; sodium chloride, 400 mg; excipient wheat middlings*.

12. * PREMIX Lda, Portugal*.

13. * Lecico P700IPM, LECICO GmbH, Germany*.

14. * DL-Methionine for Aquaculture: 99% Methionine, Evonik Nutrition & Care GmbH, Germany*.

Main ingredients were ground (below 250 μm) in a micropulverizer hammer mill (SH1; Hosokawa Micron, B.V., Doetinchem, The Netherlands). Powder ingredients and oils were then mixed according to the target formulation in a paddle mixer (RM90; Mainca, S.L., Granollers, Spain). All diets were manufactured by temperature-controlled extrusion (pellet sizes: 1.5 mm) by means of a low-shear extruder (P55; Italplast, S.r.l., Parma, Italy). Upon extrusion, all feed batches were dried in a convection oven (OP 750-UF; LTE Scientifics, Oldham, UK) for 4 h at 45°C. Formulation of experimental diets is presented in Table 1. Proximate composition analysis was conducted by the following methods: dry matter, by drying at 105°C for 24 h; ash, by combustion at 550°C for 12 h; crude protein (*N* × 6.25), by a flash combustion technique followed by gas chromatographic separation and thermal conductivity detection (LECO FP428); fat, after petroleum ether extraction, by the Soxhlet method; total phosphorus, according to the ISO/DIS 6491 method, using the vanado-molybdate reagent; gross energy, in an adiabatic bomb calorimeter (IKA).

Diets were analyzed for total AA content. Diet samples were hydrolysed in 6 M HCl at 116°C for 2 h in nitrogen-flushed glass vials. Samples were then pre-column derivatised with Waters AccQ Fluor Reagent (6-aminoquinolyl-N-hydroxysuccinimidyl carbamate) using the AccQ Tag method (Waters, USA). Analyses were done by ultra-high performance liquid chromatography (UPLC) in a Waters reversed-phase AA analysis system, using norvaline as an internal standard. During acid hydrolysis asparagine is converted to aspartate and glutamine to glutamate, so the reported values for these AA represent the sum of the respective amine and acid. Since it is partially destroyed by acid hydrolysis, tryptophan was not determined. The resultant peaks were analyzed with EMPOWER software (Waters, USA). The AA profile of the experimental diets and the relative percentage of methionine supplementation is presented in Table 2.

**Table 2:** Amino acid composition of experimental diets.

***Amino acids***	**CTRL**	**MET 0.5**	**MET 1**
		**mg AA/g DW diet**
Methionine	10.8	11.8	13.2
Arginine	39.5	39.6	39.3
Histidine	11.9	11.9	11.7
Lysine	27.9	27.8	28.4
Threonine	17.4	16.8	17.5
Isoleucine	15.9	16.1	15.8
Leucine	32.3	32.5	32.3
Valine	20.1	21.0	20.3
Phenylalanine	22.4	22.7	22.4
Cysteine	3.1	3.1	3.0
Tyrosine	16.0	16.0	15.9
Aspartic acid + Asparagine	32.2	32.6	32.5
Glutamic acid + Glutamine	70.6	71.0	70.3
Alanine	22.0	21.3	22.0
Glycine	23.0	22.4	23.4
Proline	26.7	27.4	26.4
Serine	17.8	17.3	16.9
Taurine	1.2	1.2	1.2

*Tryptophan was not analyzed*.

### Collection of blood, head kidney, and peritoneal exudates

#### Blood collection

Blood was collected from the caudal vein using heparinized syringes one part being used for hematological analysis and the remainder centrifuged at 10,000 × g 10 min at 4°C and the plasma collected, frozen on dry ice and stored at −80°C for evaluating innate humoral immune response parameters. Of the fish sampled at 2 (36 fish per group; 12 per replicate) and at 4 (36 fish per group; 12 per replicate) weeks, 9 fish from each group (3 per replicate) were used per time point for the hematological analysis. For the assessment of innate humoral immune response, plasma from all sampled fish were used, although the plasma was pooled from every 3 individuals (12 pools per treatment).

Of the fish sampled at 4, 24, and 48 h after bacterial infection (6 fish per group; 3 per replicate) the hematological analysis and the evaluation of the innate humoral immune response parameters were performed for each individual.

#### Head-kidney collection

Head-kidneys were also harvested from the 9 fish sampled at 2 and 4 weeks and used for blood collection and hematological analysis. Likewise, the head-kidneys of all fish sampled at 4, 24, and 48 h after infection were collected. After harvesting, the kidneys were immediately frozen on dry ice and stored at −80°C until processed for gene expression analysis.

#### Peritoneal exudates collection

Peritoneal cells were only collected from fish sampled at 4, 24, and 48 h post-infection (time-course), according to the procedure described by Costas et al. (22). Briefly, following fish anesthesia and bleeding by the caudal vessel, 5 ml of cold Hank's balanced salt solution (HBSS) supplemented with 30 units heparin ml^−1^ was injected into the peritoneal cavity. The peritoneal area was then slightly massaged in order to disperse peritoneal cells in the injected HBSS. The i.p. injected HBSS containing suspended cells were collected and total peritoneal leucocytes counts were performed with a hemocytometer.

### Analysis of hematological parameters

The hematological profile was conducted according to Machado et al. (17) and comprised the total white (WBC) and red (RBC) blood cells counts, as well as haematocrit (Ht) and hemoglobin (Hb; SPINREACT kit, ref. 1001230, Spain) assessments. Afterwards, the mean corpuscular volume (MCV), mean corpuscular hemoglobin (MCH) and mean corpuscular hemoglobin concentration (MCHC) were also calculated (17). Ht was not assessed in fish sampled at 4, 24, and 48 h post-infection.

Immediately after blood collection, blood smears were performed from homogenized blood and air dried. After fixation with formol-ethanol (10 of 37% formaldehyde in absolute ethanol) detection of peroxidase was carried out as described by Afonso et al. (23) in order to facilitate identification of neutrophils. Blood smears were then stained with Wright's stain (Haemacolor; Merck) Slides were examined (1,000 × ), and at least 200 leucocytes were counted and classified as thrombocytes, lymphocytes, monocytes and neutrophils. Absolute value (× 10^4^ ml^−1^) of each cell type was calculated according to the total blood WBC count.

### Analysis of innate immune response parameters

#### Lysozyme activity

Lysozyme activity was measured using a turbidimetric assay as described by Costas et al. (22). A solution of *Micrococcus lysodeikticus* (0.5 mg ml^−1^, 0.05 M sodium phosphate buffer, pH 6.2) was prepared. In triplicates, 15 μl of plasma was added to a microplate and 250 μl of the above suspension were pipetted to give a final volume of 265 μl. The reaction was carried out at 25°C and the absorbance (450 nm) was measured after 0.5 and 4.5 min in a Synergy HT microplate reader. Serial diluted, lyophilized hen egg white lysozyme (Sigma) in sodium phosphate buffer (0.05 M, pH 6.2), was used to develop a standard curve. The amount of lysozyme in the sample was calculated using the formula of the standard curve.

#### Peroxidase activity

Total peroxidase activity in plasma was measured following the procedure described by Quade and Roth (24). In triplicates, 15 μl of plasma was diluted with 135 μl of HBSS without Ca^+2^ and Mg^+2^ in flat-bottomed 96-well plates. Then, 50 μl of 20 mM 3,3′,5,5′-tetramethylbenzidine hydrochloride (TMB; Sigma) and 50 μl of 5 mM H_2_O_2_ were added. After 2 min, the color-change reaction was stopped by adding 50 μl of 2 M sulphuric acid and the optical density was read at 450 nm in a Synergy HT microplate reader. Wells without plasma were used as blanks. The peroxidase activity (units ml^−1^ plasma) was determined by defining one unit of peroxidase as that which produces an absorbance change of 1 OD.

#### Bactericidal activity

The bactericidal activity assay was performed using *Phdp* strain PP3. Bacteria were cultured in tryptic soy broth (TSB) (Difco Laboratories) supplemented with NaCl to a final concentration of 2% (w/v) (TSB-2) and exponentially growing bacteria were resuspended in sterile HBSS and adjusted to 1 × 10^6^ cfu ml^−1^. Plating serial dilutions of the suspensions onto TSA-2 plates and counting the number of cfu following incubation at 22°C confirmed bacterial concentration of the inoculum. Plasma bactericidal activity was then determined following the method described by Graham and Secombes (25) with modifications (17).

Briefly, 20 μl of plasma were added to duplicate wells of a U-shaped 96-well plate. HBSS was added to some wells instead of plasma and served as positive control. To each well, 20 μl of *Phdp* (1 × 10^6^ cfu ml^−1^) were added and the plate was incubated for 2.5 h at 25°C. 25 μl of 3-(4, 5 dimethyl-2-yl)-2,5-diphenyl tetrazolium bromide (MTT, 1 mg ml^−1^; Sigma) were then added to each well and incubated for 10 min at 25°C to allow the formation of formazan. Plates were then centrifuged at 2,000 × *g* for 10 min and the precipitate was dissolved in 200 μl of dimethyl sulfoxide (Sigma). The absorbance of the dissolved formazan resulting from the reduction of MTT in direct proportion to the number of viable bacteria present, was measured at 560 nm. Viable bacteria was expressed as percentage, calculated from the difference between the dissolved formazan in samples and the one formed in the positive controls (100%). The bactericidal activity was calculated as the percentage of non-viable bacteria.

#### Alternative complement pathway activity

Alternative complement pathway activity (ACH50) was evaluated as described by Sunyer and Tort (26). Three buffers were previously prepared: GVB (Isotonic veronal buffered saline), pH 7.3, containing 0.1% gelatin; EDTA-GVB, as previous one but containing 20 mM EDTA; and Mg-EGTA-GVB, which is GVB with 10 mM Mg^2+^ and 10 mM EGTA. Rabbit red blood cells (RaRBC; Probiologica Lda., Portugal) were washed four times in GVB and resuspended in the same buffer to a concentration of 2.5 × 10^8^ cells ml^−1^. Then, 10 μl of RaRBC suspension were added to 40 μl of serially diluted plasma in Mg-EGTA-GVB buffer in triplicates. Following an incubation time of 100 min at room temperature with continuous shaking, the reaction was stopped by adding 150 μl of cold EDTA-GVB. Samples were then centrifuged for 2.5 min at 120 × g and the extent of haemolysis was estimated by measuring the optical density of the supernatant at 414 nm. The ACH50 units were defined as the concentration of plasma inducing 50% haemolysis of RaRBC.

### Gene expression analysis

Total RNA isolation was conducted with NZY Total RNA Isolation kit (NZYTech, Lisbon, Portugal) following manufacturer's specifications. First-strand cDNA was synthesized with NZY First-Strand cDNA Synthesis Kit (NZYTech, Lisbon, Portugal). Quantitative PCR assays were performed with an Eppendorf Mastercycle ep realplex, using 1 μl of diluted cDNA (1:5 dilution) mixed with 10 μl of NZYSpeedy qPCR Master Mix and 0.4 μl (10 μM) of each specific primer in a final volume of 20 μl. cDNA amplification was carried out with specific primers (Table S1) for genes that have been selected for their involvement in immune responses and methionine metabolism (Table 3). Primers were designed with NCBI Primer Blast Tool according to known qPCR restrictions (amplicon size, Tm difference between primers, GC content and self-dimer or cross-dimer formation). Sequences encoding European seabass *tlr2, stat 3, mtor, c3zeta, ccr3, mcsf1r1*, and *cd8*β were identified after carrying out a search in the databases v1.0c seabass genome (27) and designed as previously described. S was used to analyse the efficiency of the primer pairs by calculating the slope of the regression line of the cycle thresholds (Ct) vs. the relative concentration of cDNA.

**Table 3:** Immune-related genes analyzed by real-time PCR.

**Gene**	**Acronym**	**Gene**	**Acronym**
40s Ribossomal protein (House-Keeping)	*40s*	Cluster of differentiation 8 beta	*cd8β*
Interleukin 1 β	*il1β*	Toll-like receptor 9	*tlr9*
Interleukin 8	*il8*	Toll-like receptor 2	*tlr2*
Interleukin 6	*il6*	Macrophage colony-stimulating factor 1 receptor 1	*mcsf1r1*
Transforming growth factor-beta	*tgfβ*	Matrix-metalloproteinase 9	*mmp9*
Tumor necrosis factor-alpha	*tnfα*	Complement factor 3	*c3*
Cyclo-oxygenase 2	*cox 2*	Mechanistic target of rapamycin	*mtor*
Interleukin 10	*il10*	Caspase 3	*casp 3*
C-C chemokine receptor type 3	*ccr3*	Caspase 1	*casp 1*
Chemokine CXC receptor 4	*cxcr4*	Signal transducers and activators of transcription	*stat 3*
Superoxide dismutase	*sod*	Melanocortin 2 receptor	*mc2r*
Gutathione peroxidase	*gpx*	Heat shock protein 70	*hsp70*
Hepcidin	*hep*	Heat shock protein 90	*hsp90*
Nitric oxide-inducible gene protein	*noxin*	Spermine/spermidine N (1)-acetyltransferase	*sat 1*
Major histocompatibility complex II antigen beta chain	*mhc II*	Adenosylmethionine decarboxylase 1	*amd 1*
Cluster of differentiation 3 zeta chain	*c3zeta*	

Accession number, efficiency values, annealing temperature, product length, and primers sequences are presented in Table S1. Melting curve analysis was also performed to verify that no primer dimers were amplified. The standard cycling conditions were 94°C initial denaturation for 2 min, followed by 40 cycles of 94°C denaturation for 30 s, primer annealing temperature (Table S1) for 30 s and 72°C extension for 30 s. All reactions were carried out as technical duplicates. The expression of the target genes was normalized using the expression of European seabass ribosome 40s subunit (*40s*).

### Analysis of the peritoneal leukocyte populations

Peritoneal cells were collected in fish from the time-course trial, according to the procedure described in the Peritoneal Exudates Collection section. The i.p. injected HBSS containing suspended cells was collected and total peritoneal leucocytes counts were performed with a haemocytometer. Cytospin preparations were then made with a THARMAC Cellspin apparatus and stained as indicated above for blood smears. Lymphocytes, macrophages and neutrophils in the peritoneal exudates were differentially counted, and the percentage of each cell type was established after counting a minimum of 200 cells per slide. Concentration (× 10^4^ ml^−1^) of each leucocyte type was also calculated.

### Bacterial challenge

For the bacterial challenge, *Phdp*, strain PP3, isolated from yellowtail (*Seriola quinqueradiata*; Japan) by Dr Andrew C. Barnes (Marine Laboratory, Aberdeen, UK), was used. Bacteria were routinely cultured at 22°C in tryptic soy broth (TSB) or tryptic soy agar (TSA) (both from Difco Laboratories) supplemented with NaCl to a final concentration of 2% (w/v) (TSB-2 and TSA-2, respectively) and stored at −80°C in TSB-2 supplemented with 15% (v/v) glycerol. To prepare the inoculum for injection into the fish peritoneal cavities, 100 μL of stocked bacteria were cultured overnight at 22°C on TSA-2. Exponentially growing bacteria were collected and re-suspended in sterile TSB-2 and adjusted to a final concentration of 5 × 10^4^ colony forming units (cfu) ml^−1^, as confirmed by plating the resulting cultures on TSA-2 plates and counting of cfu, and each fish inoculated intraperitoneally with 100 μl (5 × 10^3^ cfu per fish) of the bacterial suspension.

### Data analysis

All results are expressed as mean ± standard deviation (mean ± SD). Data was analyzed for normality and homogeneity of variance and, when necessary, transformed before being treated statistically. All data expressed as percentage were arcsine transformed (28). Data was analyzed by two-way ANOVA, with time and diet as factors and followed by Tukey *post-hoc* test to identify differences in the experimental treatments. All statistical analyses were performed using the computer package STATISTICA 12 for WINDOWS. The level of significance used was *P* ≤ 0.05 for all statistical tests. Sampling point 4 weeks was used as time 0 h during time-course data analysis, as they represent unstimulated animal prior to infection. The Chi-square test was performed to identify differences on the cumulative mortality among dietary treatment.

## Results

### Immune status

#### Fish growth performance

Thirty six fish per group (12/replicate) were sampled and weighted at 2 and 4 weeks after feeding with the experimental diets in order to evaluate the effect of the diets on the growth performance (Table 4). Within each group, no differences were found between replicate at any sampling point and between experimental diets in any of the growth parameters evaluated.

**Table 4:** Data on the initial weight and growth performance of European seabass sampled at 2 and 4 weeks after being fed three different diets.

	**Dietary treatments**					
**Parameters**	**CTRL**		**MET 0.5**		**MET 1**	
	**2 weeks**	**4 weeks**	**2 weeks**	**4 weeks**	**2 weeks**	**4 weeks**
Initial weight (g)	8.75 ± 1.02		8.37 ± 0.46		8.35 ± 0.48	
Final weight (g)	9.74 ± 0.58*	11.43 ± 0.33	9.48 ± 0.05*	11.37 ± 0.85	9.74 ± 0.29*	11.57 ± 0.42
Weight gain^1^ (%)	14.74 ± 11.32*	34.74 ± 11.68	15.67 ± 12.77*	38.42 ± 12.75	13.16 ± 5.92*	42.30 ± 10.52
RGR^2^ (% day^−1^)	0.95 ± 0.73	1.06 ± 0.32	0.90 ± 0.38	1.09 ± 0.43	1.11 ± 0.26	1.17 ± 0.11

*Values are presented as means ± SD (n = 36). P-values from two-way ANOVA (p ≤ 0.05). If interaction was significant, Tukey post-hoc test was used to identify differences in the experimental treatments*.

1 *Weight gain = (final weight × 100)/initial weight*.

2 *Relative Growth Rate = (e ((ln (final weight) – ln (initial weight))/days^−1^) – 1) × 100*.

*Asterisk stands for significant differences between times for the same diet*.

#### Hematology and peripheral leucocyte responses

The blood of 9 fish from each group (3 per replicate), sampled at 2 and 4 weeks, was used for evaluation of hematological parameters. The hematological profile showed few changes throughout the 2–4 weeks period, with no alteration in the haematocrit. An increase of red blood cells (RBC) numbers from 2 to 4 weeks was observed within each dietary treatment, although the hemoglobin (Hb) levels have remained unaffected. With the exception of the mean corpuscular hemoglobin concentration (MCHC), which remain unchanged between 2 and 4 weeks, all other parameters analyzed (mean corpuscular volume, MCV; mean corpuscular hemoglobin, MCH; white blood cells, WBC) decreased from 2 to 4 weeks in each diet.

Among the different diets, and despite decreasing from 2 to 4 weeks, the WBC number was increased in the diet supplemented with 1% methionine when compared to the values observed at equivalent times for the CTRL diet (Table 5), being this increase due to a greater number of neutrophils (Table 6). In fact, with respect to the concentration of each type of leukocyte analyzed in the blood, the only difference detected between the diets was a higher number of neutrophils in the blood of the fish fed with the MET 1 diet compared to those fed with the diet CTRL and MET 0.5, but no differences were observed between 2 and 4 weeks within each group. However, for thrombocytes, lymphocytes and monocytes, although they did not vary among the fish fed the different diets, there was a decrease in their number from 2 to 4 weeks within each treatment, correlating with the decrease in WBC from 2 to 4 weeks in each diet.

**Table 5:** Haematocrit, hemoglobin, mean corpuscular volume (MCV), mean corpuscular hemoglobin (MCH), mean corpuscular hemoglobin concentration (MCHC), red blood cells (RBC), and white blood cells (WBC) in European seabass fed dietary treatments during 2 and 4 weeks.

**Parameters**	**Dietary treatments**					
	**CTRL**		**MET 0.5**		**MET 1**	
	**2 weeks**	**4 weeks**	**2 weeks**	**4 weeks**	**2 weeks**	**4 weeks**
Haematocrit (%)	21.50 ± 2.60	22.63 ± 4.55	20.63 ± 3.12	22.50 ± 4.56	22.56 ± 2.17	20.43 ± 4.81
Hemoglobin (g dl)	1.09 ± 0.20	1.40 ± 0.68	1.11 ± 0.18	1.23 ± 0.40	1.16 ± 0.16	1.18 ± 0.49
MCV (μm^3^)	190.57 ± 67.55	122.44 ± 7.64	159.77 ± 16.44	126.48 ± 49.00	156.15 ± 19.13	106.09 ± 8.71
MCH (pg cell^−1^)	9.37 ± 2.34	8.64 ± 2.47	8.50 ± 1.36	6.72 ± 2.73	8.02 ± 0.98	6.21 ± 2.53
MCHC (g 100 ml^−1^)	5.33 ± 0.85	5.56 ± 1.94	5.49 ± 0.48	5.66 ± 1.74	5.15 ± 0.40	6.59 ± 2.44
RBC (× 10^6^ μl^−1^)	1.21 ± 0.29	1.83 ± 0.34	1.32 ± 0.19	1.92 ± 0.42	1.46 ± 0.14	1.95 ± 0.39
WBC (× 10^4^ μl^−1^)	7.83 ± 1.71	4.67 ± 0.80	9.09 ± 3.28	5.73 ± 0.65	9.66 ± 1.59	6.40 ± 1.45
**Two-way ANOVA**						
**Parameters**				**Diet**		
	**Time**	**Diet**	**Time** × **Diet**	**CTRL**	**MET 0.5**	**MET 1**
Haematocrit	Ns	ns	ns	–	–	–
Hemoglobin	ns	ns	ns	–	–	–
MCV	<0.001	ns	ns	–	–	–
MCH	0.032	ns	ns	–	–	–
MCHC	ns	ns	ns	–	–	–
RBC	<0.001	ns	ns	–	–	–
WBC	<0.001	0.032	ns	B	AB	A

*Values are presented as means ± SD (n = 9). P-values from two-way ANOVA (p ≤ 0.05). If interaction was significant, Tukey post-hoc test was used to identify differences in the experimental treatments. Different capital letters indicate differences among diets regardless time*.

**Table 6:** Absolute values of peripheral blood leucocytes (thrombocytes, lymphocytes, monocytes, and neutrophils) of European seabass fed dietary treatments during 2 and 4 weeks.

**Parameters**	**Dietary treatments**					
	**CTRL**		**MET 0.5**		**MET 1**	
	**2 weeks**	**4 weeks**	**2 weeks**	**4 weeks**	**2 weeks**	**4 weeks**
Thrombocytes (× 10^4^ μl^−1^)	4.16 ± 1.05	2.93 ± 0.50	4.99 ± 2.19	2.71 ± 0.88	4.68 ± 1.03	2.96 ± 0.81
Lymphocytes (× 10^4^ μl^−1^)	3.63 ± 0.89	1.58 ± 0.40	4.88 ± 2.20	2.07 ± 0.52	4.40 ± 1.21	2.52 ± 0.91
Monocytes (× 10^4^ μl^−1^)	0.23 ± 0.08	0.12 ± 0.05	0.23 ± 0.14	0.15 ± 0.09	0.25 ± 0.10	0.20 ± 0.13
Neutrophils (× 10^4^ μl^−1^)	0.06 ± 0.07	0.03 ± 0.02	0.02 ± 0.02	0.08 ± 0.11	0.12 ± 0.04	0.17 ± 0.12
**Two-way ANOVA**						
**Parameters**				**Diet**		
	**Time**	**Diet**	**Time** × **Diet**	**CTRL**	**MET 0.5**	**MET 1**
Thrombocytes	<0.001	ns	ns	–	–	–
Lymphocytes	<0.001	ns	ns	–	–	–
Monocytes	0.018	ns	ns	–	–	–
Neutrophils	ns	0.004	ns	B	B	A

*Values are presented as means ± SD (n = 9). P-values from two-way ANOVA (p ≤ 0.05) (n = 9). If interaction was significant, Tukey post-hoc test was used to identify differences in the experimental treatments. Different capital letters indicate differences among diets regardless time*.

Thus, while the decrease in the number of WBC from 2 to 4 weeks observed in the fish fed with each of the diets was due to the decrease in the number of thrombocytes, monocytes and lymphocytes, the highest number of WBC observed in fish fed with the diet supplemented with 1% methionine was exclusively due to a higher number of neutrophils, suggesting the stimulation of an inflammatory response by methionine supplementation.

#### Humoral innate immune response

For the evaluation of the innate humoral response, 36 fish were collected from each experimental group (12 per replicate) and, for reasons of quantity limitation, the plasma from each 3 fish was pooled. Humoral innate immune parameters assessed in plasma are presented in Table 7.

**Table 7:** Plasma lysozyme, peroxidase, ACH50, and bactericidal activities of European seabass fed dietary treatments during 2 and 4 weeks.

**Parameters**	**Dietary treatments**					
	**CTRL**		**MET 0.5**		**MET 1**	
	**2 weeks**	**4 weeks**	**2 weeks**	**4 weeks**	**2 weeks**	**4 weeks**
Lysozyme (μg mg ml^−1^)	3.51 ± 0.92^a^*	1.08 ± 0.91	1.62 ± 0.95^b^	0.89 ± 0.61	1.39 ± 0.84^b^	0.97 ± 0.41
Peroxidase (units ml^−1^)	124.45 ± 32.04	89.80 ± 17.36	126.56 ± 201.13	132.42 ± 38.96	129.04 ± 48.06	118.52 ± 40.32
Bactericidal activity (%)	30.39 ± 7.16	25.82 ± 10.81	30.13 ± 6.57	22.04 ± 11.65	41.15 ± 7.52	25.86 ± 4.23
ACH50 (units ml^−1^)	74.71 ± 24.46	197.32 ± 60.91	78.19 ± 21.27	120.47 ± 42.80	96.85 ± 28.88	119.00 ± 37.06
**Two-way ANOVA**						
**Parameters**				**Diet**		
	**Time**	**Diet**	**Time** × **Diet**	**CTRL**	**MET 0.5**	**MET 1**
Lysozyme	<0.001	<0.001	<0.001	A	A	B
Peroxidase	ns	ns	ns	–	–	–
Bactericidal activity	<0.001	0.002	ns	AB	B	A
ACH50	0.007	ns	ns	–	–	–

*Values are presented as means ± SD (n = 12). P-values from two-way ANOVA (p ≤ 0.05). If interaction was significant, Tukey post-hoc test was used to identify differences in the experimental treatments. Different lowercase letters stand for significant differences among dietary treatments for the same time, while asterisk stands for significant differences between times for the same diet. Different capital letters indicate differences among diets regardless time*.

Two weeks after the beginning of feeding of the experimental diets, plasma of fish fed diets supplemented with methionine (MET 0.5 and MET 1) presented lower lysozyme concentration than that found in the plasma of fish fed with the control diet. Furthermore, a decrease from 2 to 4 weeks was observed in the lysozyme concentration for all diets, although only statistically significant for fish fed CTRL. Such decrease of lysozyme concentration could explain the reduction of the total bactericidal activity with time for all diets. Plasma bactericidal activity was found to be higher in fish fed MET 1 relative to those fed MET 0.5

Regarding the alternative complement pathway, there were no differences in activity between the different treatments, although its activity increased from 2 to 4 weeks in fish fed any of the diets.

#### Head-kidney gene expression

To evaluate the expression of genes related to immune response and methionine metabolism role in immune response (Table 3), cDNA was isolated from head-kidneys collected from 9 fish from each group (3 per replicate).

High variability in the expression of many of the analyzed genes was observed, with statistically significant differences in the expression of the genes coding for IL-1b, Noxin, CD8β, Caspase-3, Melanocortin 2 receptor, and Spermine/spermidine N (1)-acetyltransferase.

**Figure 2:**
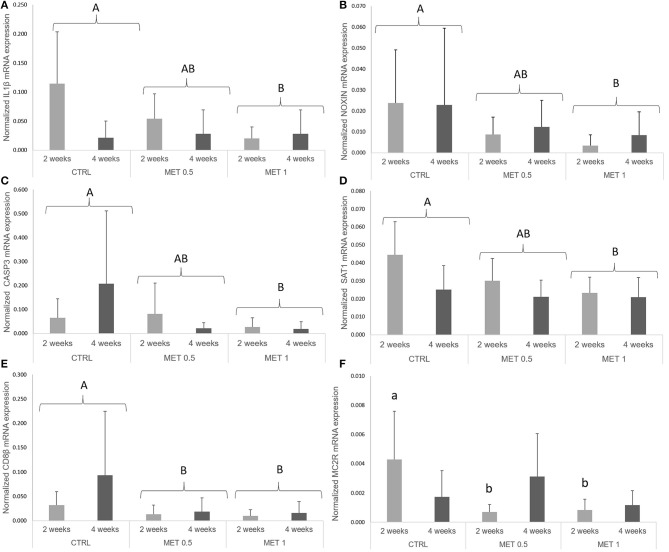
Quantitative expression **(A)** interleukin 1 β, **(B)** nitric oxide-inducible gene protein, **(C)** caspase 3, **(D)** spermine/spermidine N (1)-acetyltransferase, **(E)** cluster of differentiation 8 beta and **(F)** melanocortin 2 receptor genes in the head-kidney of European seabass fed dietary treatments during 2 and 4 weeks. Values are presented as means ± SD (*n* = 9). *P*-values from two-way ANOVA (*p* ≤ 0.05). If interaction was significant, Tukey *post-hoc* test was used to identify differences in the experimental treatments. Different lowercase letters stand for significant differences among dietary treatments for the same time. Different capital letters indicate differences among diets regardless time.

The normalized *sat1* expression level showed a decrease between both sampling times (Table S2). Moreover, *il1*β (Figure 2A), *noxin* (Figure 2B), *casp3* (Figure 2C), and *sat1* (Figure 2D) mRNA expression level was lower in fish fed MET 1 than in fish fed CTRL, while fish fed MET 0.5 and MET 1 presented lower *cd8*β (Figure 2E) expression levels than fish fed CTRL dietary treatment. Fish fed MET 0.5 and Met 1 presented decreased *mc2r* (Figure 2F) transcripts compared to fish fed CTRL after 2 weeks of feeding. The data regarding gene expression during the feeding trial is presented in Table S2 as Supplementary Data.

### Bacterial challenge

**Figure 3:**
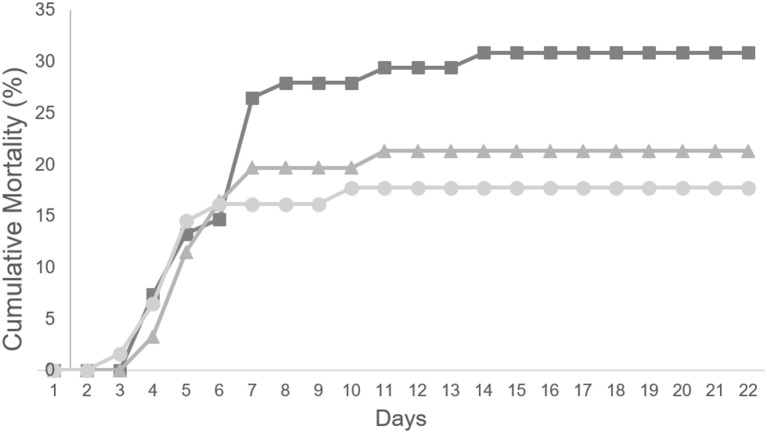
Cumulative mortality (%) of European seabass fed CTRL (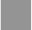), MET 0.5 (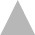) and MET 1 (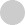) dietary treatments for 4 weeks and subsequently infected with *Phdp* (*n* = 60).

To evaluate a possible protective effect of a diet supplemented with methionine during a bacterial infection, 60 fish from each group (20/replicate) were inoculated with *Phdp* and their mortality followed for 3 weeks (Figure 3). Fish fed any of the diets supplemented with methionine, MET0.5 and MET1, showed lower mortality than fish fed the CTRL diet, with a relative percentage survival (RPS) to fish fed the CTRL diet of 32 and 43%, respectively.

Although no statistically significant differences were detected between the RPS observed between the experimental groups, this is most probably due to the fact that the number of fish per group calculated (power analysis) assuming that the supplemented diets would promote an RPS to to fish fed the CTRL diet higher than 60%.

### Infection response

To examine the influence that methionine supplementation may have on the initial inflammatory response following *Phdp* infection, samples of blood, head kidney, and peritoneal exudates were collected at 4, 24, and 48 h post-infection from fish of each experimental group (6 fish from each experimental diet by time-point). Sampling point 4 weeks was used as time 0 h during time-course data analysis, as they represent unstimulated animal prior to infection. Thus, the collected samples were used to analyze whether the diets supplemented with methionine, compared to the control diet, caused hematological alterations, influenced the innate immune response and the expression of genes in the head kidney as well as induced changes in the intraperitoneal leukocyte populations.

#### Hematology and peripheral leucocyte responses

The concentration of RBC in the fish blood was higher at 4 h after infection with *Phdp* regardless of the dietary treatment, with no effect of methionine supplementation observed, since no differences were observed between the experimental groups (Table 8). Regarding the hemoglobin concentration and the MCH index, no changes were observed over time within each experimental group nor between fish fed the different diets. Similarly to the RBCs, an increase in the number of WBCs in fish blood was also observed 48 h after infection when compared to the number of WBCs at earlier times. However, in a manner different from that observed for the concentration of RBC, it appears that methionine supplementation had an influence on the number of WBC in the blood of the fish fed with the supplemented diets, since their number is increased compared to the number of WBC in the blood of fish fed the control diet (Table 9). As already noted before infection, this increase in the number of WBC in the blood of fish fed diets supplemented with methionine seems to be due to the increase in circulating neutrophils since the number of these cells not only increases over time within any experimental group, as it is increased relative to that observed in the blood of the fish that were fed the control diet, while no differences between treatments were detected relative to the number of other leukocytes. However, within each treatment, and as observed for neutrophils, the number of thrombocytes, lymphocytes and monocytes were increased in fish blood after 48 h after infection compared to the time immediately before infection (0 h), but: (i) in the case of thrombocytes, the increase was observed at 4 h after infection, remaining high and without variation until 48 h: (ii) in the case of lymphocytes, there was a decrease in the initial times post-infection (4 and 24 h) increasing their number at 48 h; and (iii) in the case of monocytes, their number remained constant until 24 h, increasing at 48 h.

**Table 8:** Hemoglobin, mean corpuscular hemoglobin (MCH), red blood cells (RBC), and white blood cells (WBC) in European seabass fed dietary treatments at 4 weeks (0 h), 4, 24, and 48 h after infection.

**Parameters**	**Dietary treatments**											
	**CTRL**				**MET 0.5**				**MET 1**			
	**0 h**	**4 h**	**24 h**	**48 h**	**0 h**	**4 h**	**24 h**	**48 h**	**0 h**	**4 h**	**24 h**	**48 h**
Hemoglobin (g dl)	1.40 ± 0.68	1.18 ± 0.26	0.94 ± 0.20	1.46 ± 0.30	1.23 ± 0.40	1.01 ± 0.22	0.93 ± 0.21	1.16 ± 0.23	1.18 ± 0.49	1.40 ± 0.47	1.23 ± 0.34	1.27 ± 0.14
MCH (pg cell^−1^)	8.64 ± 2.47	6.81 ± 1.35	4.91 ± 0.96	6.43 ± 0.84	6.51 ± 1.82	6.44 ± 1.52	4.78 ± 0.63	5.02 ± 1.06	6.21 ± 2.53	7.92 ± 3.31	6.37 ± 2.47	6.04 ± 1.45
RBC (× 10^6^ μl^−1^)	1.83 ± 0.34	1.76 ± 0.32	1.93 ± 0.38	2.26 ± 0.30	1.92 ± 0.42	1.57 ± 0.15	1.95 ± 0.33	2.35 ± 0.37	1.95 ± 0.39	1.83 ± 0.16	2.00 ± 0.32	2.16 ± 0.26
WBC (× 10^4^ μl^−1^)	4.67 ± 0.80	6.52 ± 1.11	5.60 ± 1.16	8.53 ± 1.92	5.73 ± 0.65	6.28 ± 0.79	6.40 ± 1.06	10.17 ± 1.28	6.40 ± 1.45	6.52 ± 1.12	6.48 ± 0.64	8.95 ± 1.06
**Two-way ANOVA**									
				**Time**				**Diet**		
**Parameters**	**Time**	**Diet**	**Time** × **Diet**	**0 h**	**4 h**	**24 h**	**48 h**	**CTRL**	**MET 0.5**	**MET 1**
Hemoglobin	ns	ns	Ns	–	–	–	–	–	–	–
MCH	ns	ns	ns	–	–	–	–	–	–	–
RBC	<0.001	ns	ns	B	B	AB	A	–	–	–
WBC	<0.001	0.035	ns	B	B	B	A	B	A	A

*Values are presented as means ± SD (n = 6). P-values from two-way ANOVA (p ≤ 0.05). If interaction was significant, Tukey post-hoc test was used to identify differences in the experimental treatments. Different capital letters indicate differences among times regardless diets and among diets regardless time*.

**Table 9:** Absolute values of peripheral blood leucocytes (i.e., thrombocytes, lymphocytes, monocytes, and neutrophils) of European seabass fed dietary treatments at 4 weeks (0 h), 4, 24, and 48 h after infection.

**Parameters**	**Dietary treatments**											
	**CTRL**				**MET 0.5**				**MET 1**			
	**0 h**	**4 h**	**24 h**	**48 h**	**0 h**	**4 h**	**24 h**	**48 h**	**0 h**	**4 h**	**24 h**	**48 h**
Thrombocytes (× 10^4^ μl^−1^)	2.93 ± 0.50	4.49 ± 0.69	3.69 ± 0.91	3.89 ± 0.40	2.71 ± 0.88	4.53 ± 0.88	4.15 ± 0.56	4.56 ± 0.45	2.96 ± 0.81	3.96 ± 0.80	3.79 ± 0.41	4.27 ± 0.48
Lymphocytes (× 10^4^ μl^−1^)	1.58 ± 0.40	1.49 ± 0.32	1.23 ± 0.43	3.30 ± 1.39	2.07 ± 0.52	1.09 ± 0.24	1.24 ± 0.36	4.16 ± 0.58	2.52 ± 0.91	1.78 ± 0.56	1.56 ± 0.25	3.42 ± 0.70
Monocytes (× 10^4^ μl^−1^)	0.12 ± 0.05	0.06 ± 0.03	0.15 ± 0.06	0.54 ± 0.27	0.15 ± 0.09	0.10 ± 0.04	0.19 ± 0.11	0.40 ± 0.15	0.20 ± 0.13	0.25 ± 0.09	0.18 ± 0.06	0.48 ± 0.13
Neutrophils (× 10^4^ μl^−1^)	0.03 ± 0.02	0.47 ± 0.33	0.62 ± 0.15	0.89 ± 0.42	0.08 ± 0.11	0.74 ± 0.21	0.90 ± 0.22	1.10 ± 0.91	0.17 ± 0.12	0.67 ± 0.10	0.96 ± 0.32	0.91 ± 0.38
**Two-way ANOVA**										
				**Time**				**Diet**		
**Parameters**	**Time**	**Diet**	**Time** × **Diet**	**0 h**	**4 h**	**24 h**	**48 h**	**CTRL**	**MET 0.5**	**MET 1**
Thrombocytes	<0.001	ns	ns	B	A	A	A	–	–	–
Lymphocytes	<0.001	ns	ns	B	C	C	A	–	–	–
Monocytes	<0.001	ns	ns	B	B	B	A	–	–	–
Neutrophils	<0.001	0.045	ns	C	B	AB	A	B	A	A

*Values are presented as means ± SD (n = 6). P-values from two-way ANOVA (p ≤ 0.05). If interaction was significant, Tukey post-hoc test was used to identify differences in the experimental treatments. Different capital letters indicate differences among times regardless diets and among diets regardless time*.

#### Analysis of the peritoneal leucocytes responses

Total and differential peritoneal leucocytes counts were only performed in infected fish with the aim to assess cell migration dynamics to the inflammation site following bacterial injection, and are presented in Table 10. Fish fed MET 1 displayed a higher leucocyte population in the peritoneal cavity at 48 h than fish fed CTRL and MET 0.5, matching with the larger number of lymphocytes, macrophages and neutrophils at the same time compared to those fed with the other diets, although, due to the high variability observed in the macrophage count, no statistically significant difference was detected in the number of this type of cells. In fact, in general, an increase of all leukocyte populations over time was observed in the peritoneal cavity of fish fed the diet with higher methionine supplementation, which supports the occurrence of a stronger local inflammatory response after the intraperitoneal infection with *Phdp* in fish fed with this diet.

**Table 10:** Absolute values of peritoneal leucocytes, lymphocytes, macrophages, and neutrophils of European seabass fed dietary treatments at 4, 24, and 48 h after infection.

**Parameters**	**Dietary treatments**								
	**CTRL**			**MET 0.5**			**MET 1**		
	**4 h**	**24 h**	**48 h**	**4 h**	**24 h**	**48 h**	**4 h**	**24 h**	**48 h**
Leucocytes (× 10^4^ μl^−1^)	8.70 ± 3.59	5.75 ± 1.82	10.90 ± 2.57^b^	6.68 ± 2.03	14.40 ± 6.28	10.88 ± 1.51^b^	12.62 ± 5.99*	10.57 ± 4.03*	22.00 ± 4.57^a^
Lymphocytes (× 10^4^ μl^−1^)	2.37 ± 1.04	1.73 ± 0.88	1.45 ± 0.42	1.80 ± 1.20	1.63 ± 0.58	2.55 ± 1.07	2.28 ± 1.31*	4.85 ± 1.75	5.07 ± 2.15
Macrophages (× 10^4^ μl^−1^)	6.58 ± 2.08	5.51 ± 2.80	5.25 ± 1.61	4.26 ± 3.43	3.00 ± 0.86	4.06 ± 1.76	4.12 ± 1.07	8.83 ± 1.52	8.16 ± 2.71
Neutrophils (× 10^4^ μl^−1^)	1.77 ± 0.73	0.88 ± 0.37^b^	2.02 ± 0.53^b^	1.53 ± 0.44	3.37 ± 1.37^a^	1.69 ± 0.19^b^	3.52 ± 1.75	3.10 ± 1.39^a^	4.35 ± 1.64^a^
**Two-way ANOVA**									
**Parameters**				**Time**			**Diet**		
	**Time**	**Diet**	**Time** × **Diet**	**4 h**	**24 h**	**48 h**	**CTRL**	**MET 0.5**	**MET 1**
Leucocytes	<0.001	<0.001	0.003	B	B	A	B	B	A
Lymphocytes	0.028	ns	0.034	B	A	AB	–	–	–
Macrophages	<0.001	0.005	ns	C	B	A	B	B	A
Neutrophils	ns	<0.001	0.025	–	–	–	B	B	A

*Values are presented as means ± SD (n = 6). P-values from two-way ANOVA (p ≤ 0.05). If interaction was significant, Tukey post-hoc test was used to identify differences in the experimental treatments. Different lowercase letters stand for significant differences among dietary treatments for the same time while different symbols stands for significant differences between times for the same diet. Different capital letters indicate differences among times regardless diets and among diets regardless time*.

#### Plasma humoral responses

Fish fed MET 1 showed higher lysozyme activity at 24 and 48 h whereas fish fed MET 0.5 presented an increased activity at 48 h after infection compared to fish fed CTRL. Moreover, fish fed the CTRL dietary treatment showed higher lysozyme concentration at 48 h than at 0 and 4 h after infection, while fish fed MET 0.5 and MET 1 presented higher values at 24 h than at 4 or 0 and 4 h, respectively (Table 11). Peroxidase activity decreased at 4 h compared to the other sampling points regardless dietary treatment whereas fish fed MET 0.5 showed an increased peroxidase activity compared to fish fed CTRL and MET 1 diets regardless time (Table 11). Bactericidal activity was found to increase after injection and a peak was found at 48 h. Lastly, MET 0.5 displayed higher ACH50 levels at 24 h in comparison to the other dietary treatments.

**Table 11:** Plasma lysozyme, peroxidase, ACH50, and bactericidal activities of European seabass fed dietary treatments at 4 weeks (0 h), 4, 24, and 48 h after infection.

**Parameters**	**Dietary treatments**											
	**CTRL**				**MET 0.5**				**MET 1**			
	**0 h**	**4 h**	**24 h**	**48 h**	**0 h**	**4 h**	**24 h**	**48 h**	**0 h**	**4 h**	**24 h**	**48 h**
Lysozyme (μg mg ml^−1^)	1.08 ± 0.91*	0.27 ± 0.09*	2.22 ± 1.66^b^*	2.88 ± 2.65^b^	0.89 ± 0.61*	2.22 ± 2.77*	5.13 ± 3.05^a^	3.12 ± 1.99^ab^*	0.97 ± 0.41*	1.95 ± 0.96*	5.76 ± 2.49^a^	4.45 ± 2.39^a^*
Peroxidase (units ml^−1^)	89.80 ± 17.36	88.49 ± 14.25	123.17 ± 37.15	84.50 ± 33.55	132.42 ± 38.96	87.00 ± 17.28	121.85 ± 32.68	122.80 ± 57.29	118.52 ± 40.32	93.21 ± 17.49	113.71 ± 25.79	118.12 ± 49.96
Bactericidal activity (%)	25.82 ± 10.81	33.44 ± 6.47	39.89 ± 6.23	40.62 ± 5.44	22.04 ± 11.65	34.43 ± 6.77	36.63 ± 7.22	43.05 ± 8.88	25.86 ± 4.23	39.71 ± 5.27	36.61 ± 5.84	46.11 ± 6.18
ACH50 (units ml^−1^)	197.32 ± 60.91	216.43 ± 150.22	85.76 ± 22.59^b^	74.44 ± 24.31	120.47 ± 42.80*	148.57 ± 152.43*	540.12 ± 206.53^a^	162.93 ± 122.60*	119.00 ± 37.06	141.22 ± 85.69	78.00 ± 11.69^b^	47.14 ± 9.05
**Two-way ANOVA**											
**Parameters**				**Time**				**Diet**			
	**Time**	**Diet**	**Time** × **Diet**	**0 h**	**4 h**	**24 h**	**48 h**	**CTRL**	**MET 0.5**	**MET 1**
Lysozyme	<0.001	ns	0.008	B	B	A	A	–	–	–
Peroxidase	0.0135	0.039	ns	A	B	A	A	B	A	AB
Bactericidal activity	<0.001	ns	ns	C	B	ABC	A	–	–	–
ACH50	ns	0.005	<0.001	–	–	–	–	AB	A	B

*Values are presented as means ± SD (n = 6). P-values from two-way ANOVA (p ≤ 0.05). If interaction was significant, Tukey post-hoc test was used to identify differences in the experimental treatments. Different lowercase letters stand for significant differences among dietary treatments for the same time different symbols stands for significant differences between times for the same diet. Different capital letters indicate differences among times regardless diets and among diets regardless time*.

#### Head-kidney gene expression

To evaluate the expression of genes related to immune response and methionine metabolism role in the inflammatory response (Table 3), cDNA was isolated from head-kidneys collected from 6 fish from each group (3 per replicate).

In response to infection with *Phdp, mmp9* and *cox2* expression levels increased from 0 to 4 h. Improved expression, relative to 0 h was also observed at 24 h for *il8, casp1, hep*, and *hsp70* and for *il10, m2cr*, and *noxin* at 48 h. *Il1*β and *mtor* presented improved expression at 4 and 24 h compared to 0 h, whereas both *c3* and *stat3* were up-regulated at 24 and 48 h relative to 0 h. At 0 and 4 h, *tlr9* and *hsp90* expression levels were lower than at 24 h, while *mhcII* presented decreased values at 0 and 4 h relative to 24 and 48 h (Table S3). Both *tlr2* and *il6* expression levels were found higher at 24 h relative to 0 and 48 h and 0, 4, and 48 h, respectively. Finally, n*oxin, cox2* and *cxcr4* increased at 24 h compared to all other sampling times (Table S3).

**Figure 4:**
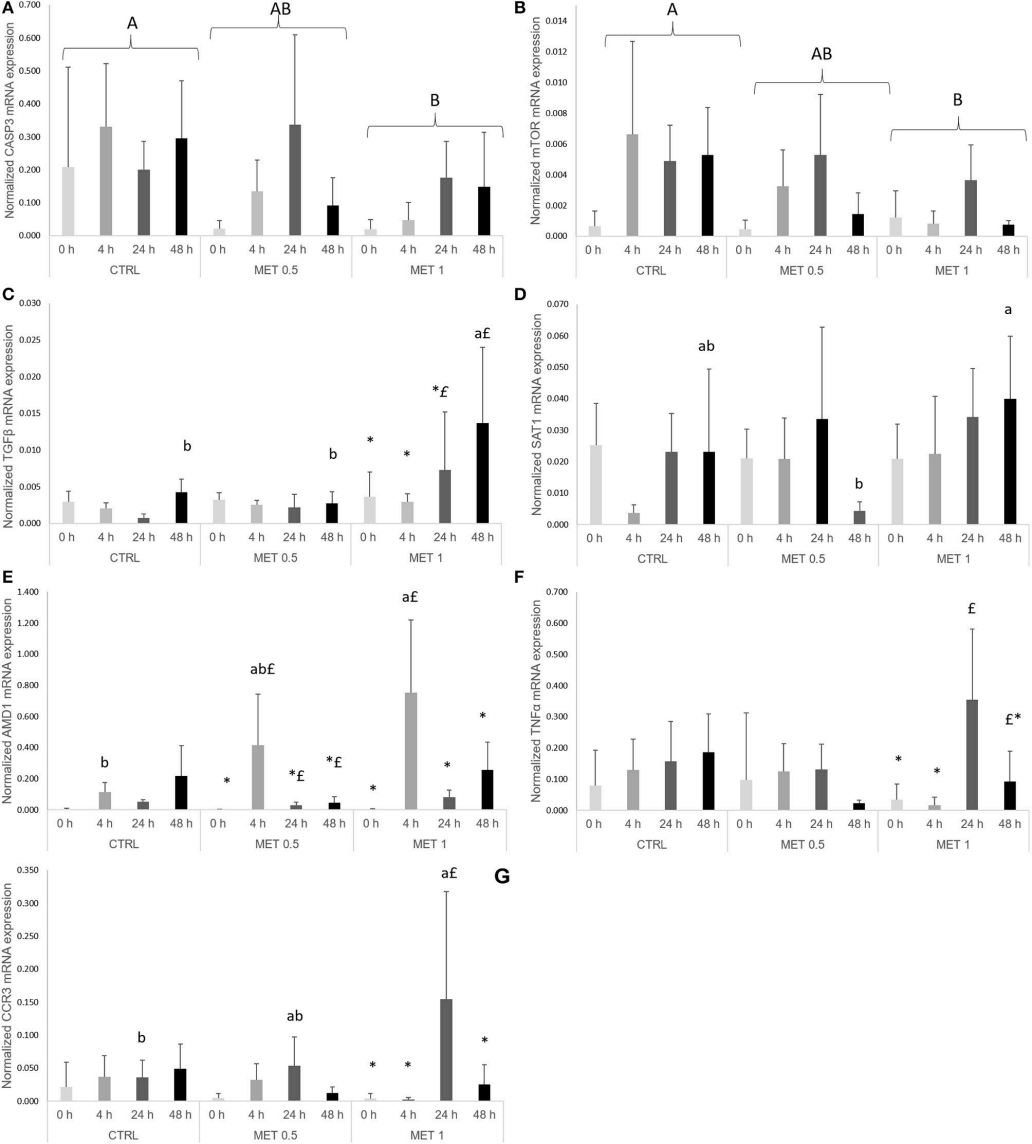
Quantitative expression of: **(A)** caspase 3, **(B)** mechanistic target of rampamycin, **(C)** transforming growth factor-beta, **(D)** spermine/spermidine N (1)-acetyltransferase, **(E)** adenosylmethionine decarboxylase 1, **(F)** tumor necrosis factor-alpha and **(G)** c-c chemokine receptor type 3 genes in the head-kidney of European seabass fed dietary treatments at 4 weeks (0 h), 4, 24, and 48 h after peritoneal infection with *Phdp*. Values are presented as means ± SD (*n* = 6). *P*-values from two-way ANOVA (*p* ≤ 0.05). If interaction was significant, Tukey *post-hoc* test was used to identify differences in the experimental treatments. Different lowercase letters stand for significant differences among dietary treatments for the same time while different symbols stand for significant differences between times for the same diet. Different capital letters indicate differences among times regardless diets and among diets regardless time.

A dietary effect was observed for *casp3*, as mRNA levels decreased in fish fed MET 1 compared to fish fed CTRL (Figure 4A). Moreover, *mtor* was found to be higher in fish fed CTRL in relation to fish fed MET 1 (Figure 4B). Fish fed MET 1 showed higher *tgf*β expression levels than fish fed CTRL and MET 0.5 dietary treatments at 48 h, while an increase in time was observed for the same dietary treatment with higher levels at 48 h than at 0 and 4 h after infection (Figure 4C). *Sat1* expression level was higher in fish fed MET 1 than those fed MET 0.5 (Figure 4D), while *amd1* transcripts increased in fish fed MET 1 relatively to fish fed CTRL at 4 h. Also, fish fed MET 1 presented an improved *amd1* expression level at 4 h in comparison to all remaining times, whereas fish fed MET 0.5 increased *amd1* transcripts at 4 h relatively to 0 h(Figure 4E). Specifically for fish fed MET 1, *tnf*α mRNA expression was higher at 24 h than at 0 and 4 h (Figure 4F), while *ccr3* expression level augmented in fish fed MET 1 relative to fish fed CTRL at 24 h and also compared to the remaining times (Figure 4G). All data regarding gene expression are presented in Table S3 as Supplementary Data.

## Discussion

The modulatory effect of dietary methionine supplementation on the European seabass immune status was here evaluated at two different levels and for two different feeding periods. A leukopenia together with a thrombocytopenia, lymphopenia and monocytopenia was observed between the two sampling times. This cell decline was accompanied by a decrease of plasma total bactericidal activity and a reduction of spermine/spermidine N (1)–acetyltransferase (SAT1) mRNA expression, a rate-limiting enzyme involved in the regulation of the intracellular concentration of polyamines. Previous works demonstrated that methionine was able to improve the European seabass immune response in the absence of a stimulus after a 15 days feeding period by presenting higher peripheral leucocytes and neutrophils concentration, improved plasma complement levels (17) and higher head-kidney *c3* mRNA expression (21). In accordance, the present study showed that methionine supplementation at the highest level led to a significant increment of total circulating leucocytes and neutrophils numbers, regardless of feeding time. Through the aminopropylation route, decarboxylated SAM, derived from methionine, is used as an aminopropyl donor to polyamine production (9). This role of methionine in polyamine synthesis may explain the enhanced leucocyte response, with a particular emphasis in neutrophils proliferation, observed in the absence of stimuli and without evidences of cell activation (e.g., neutrophils degranulation). In fact, fish fed either MET 0.5 or MET 1 dietary treatment presented a decrease in the concentration of plasmatic lysozyme after 2 weeks of feeding. This hypothesis is further supported by the down-regulation of genes encoding several pro-inflammatory indicators, such as the pro-inflammatory cytokine *il1*β, the induced gene protein of nitric oxide *noxin, casp3* with central role in cell apoptosis, as well as the transmembrane glycoprotein *cd8*β that serves as a co-receptor for the T-cell receptors. Additionally, the expression of *sat1*, known to be highly regulated by polyamines, was reduced by methionine supplementation which can be understood as a strategy to avoid non-specific deleterious effects in host tissues, as a negative feedback mechanism (29). Dietary methionine input is also recognized as a key factor that can increase methylation of specific genes, theoretically repressing them. DNA methylation is catalyzed by DNA methyltransferases that transfer methyl groups from SAM to cytosine in a specific cytosine-guanine (*CpG*) and that might be enough to change gene expression. Because DNA methyltranferases reaction is dependent on the supply of SAM and the removal of S-adenosylhomocysteine (SAH), the SAM:SAH ratio has been proposed as a “methylation ratio” (8). Moreover, Zhang (30) reviewed that due to the circular nature of methionine cycle and the complexity of the methylation reactions, the mechanisms by which methionine affects DNA methylation are poorly understood and likely to be highly dependent of tissue, animal life stage and gene region.

Methionine also plays important roles in the control of inflammatory processes, being involved in the reduction of reactive oxygen species (ROS) and protecting cells from oxidative stress through GSH metabolism (1). In the present study, the enhanced leucocyte proliferation together with lower gene expression of pro-inflammatory indicators observed at the highest methionine supplementation level tested appear to indicate that increasing methionine dietary content may improve European seabass immune status without triggering an inflammatory response. In poultry, methionine showed clear evidences of immune-stimulatory capacities, improving both humoral and cell immune responses (16, 31) while supplementation of dietary methionine enhanced platelet and leucocyte counts of male cotton rats (*Sigmodon hispidus*) (32). Besides our previous work, in which methionine-supplemented diets increased peripheral leucocytes abundance in the absence of immune stimulation (17), few more studies have focused on methionine as a health-promoting additive in aquafeeds. An increase of leucocytes concentration was observed in juvenile Jian carp fed graded levels of methionine hydroxyl analog, a synthetic methionine source, which resulted in increased survival rate after injection with *Aeromonas hydrophila* (18).

The enhanced immune status observed in the present study translated in a clear trend for increased disease resistance against *Phdp* despite the non-significant statistical result. The immune response was indeed boosted upon infection, as observed by the increased number of all peripheral leucocyte types and improved macrophages recruitment to the inflammatory focus, regardless of dietary treatment. These leucocytes migration dynamics were supported by an up-regulation of numerous pro-inflammatory genes, such as interleukins and chemokines, cell markers and receptors, transcription factors and cell stress proteins. More importantly, the enhanced immune defenses observed at the end of the feeding trial in fish fed methionine-supplemented diets were triggered by infection, as similar results were observed by Machado et al. (17) for European seabass stimulated with inactivated *Phdp* after a 15 days feeding period, and for Jian carp (18) and juvenile yellow catfish (*Pelteobagrus fulvidraco*) (33) injected with *Aeromonas hydrophila* and fed for 60 days and 11 weeks, respectively. Similar outcomes have been reported for poultry and mammals where methionine supplementation improved chicken cellular and humoral immune mechanisms in response to Newcastle disease virus (15) and partially alleviated the depression in performance caused by aflatoxin B1 in pigs (34).

In the present study, the effect of dietary methionine supplementation seems to work in a dose-response manner in terms of cell recruitment. Indeed, fish fed MET 0.5 showed higher mobilization of neutrophils to the peritoneal cavity than fish fed CTRL dietary treatment at 24 h, while fish fed MET 1 presented an increased concentration of leucocytes and neutrophils at the inflammatory focus compared to the other dietary groups at 24 and 48 h. This improved cell migration dynamics is further supported by an increased number of total peritoneal leucocytes, lymphocytes and macrophages over time, which was not observed in fish fed CTRL or MET 0.5 dietary treatments. Moreover, plasma peroxidase, lysozyme and ACH50 activities were enhanced in general by dietary methionine surplus, probably as a result of an improved activation of phagocytic cells and better development of an inflammatory response (35), a fact also observed in previous works (17, 18). This improved cell-mediated response was also accompanied by *sat1* up-regulation, as well as higher expression of the chemokine receptor *ccr3* and the multifunctional cytokine *tgf*β. *ccr3* is a receptor for multiple inflammatory/inducible CC chemokines modulating monocytes migration and other cell types, such as NK cells and dendritic cells (36). Differently, *tgf*β is produced by leucocytes and is responsible for inducing transcription of different target genes related to cell differentiation, chemotaxis, proliferation, and activation of many immune cells (37). Still, a significant reduced expression of *casp3*, essential for processes associated with the formation of apoptotic bodies, supports the role of methionine on the control of inflammation and apoptotic mechanisms (38). *mtor*, regulated by nutrients [e.g., methionine (39)], energy levels, and growth factors (40, 41), encodes a kinase that regulates key cellular functions linked to the promotion of cell growth and metabolism. *mtor* mRNA levels were reduced by methionine supplementation which can be understood as a strategy to control the boosted inflammatory response described above.

The broad range of pathways in which methionine participates may have contributed to the results here described, underpinning the proposed beneficial effect of dietary methionine supplementation on seabass immune status after a 4 weeks feeding period, while improving fish response mechanisms to an infection insult. Several studies have already demonstrated the ability of dietary supplementation of specific AA in mammals (including humans) and birds to improve immune status, stress response, reducing mortality and its practical use in industry (5, 15). On the other hand, few works have been focused on AA dietary supplementation and fish immune mechanism (17–19, 21). Further studies on polyamine and cytokine protein quantification should be considered to support these hypotheses and more confidently characterize methionine role during the inflammatory response. Nonetheless, mortality results ultimately corroborate the positive effect of methionine supplementation.

In conclusion, results from the present study clearly indicate that methionine dietary supplementation could be an important nutritional startegy for fish health management as it improved European seabass cellular immune status without triggering pro-inflammatory indicators. Furthermore, it was shown that this enhanced immune status translates into an improved inflammatory response against *Phdp*, as higher cellular differentiation/proliferation and recruitment to the inflammatory focus was observed, as well as improved plasma humoral immune parameters together with a modulation of key immune-related genes. Lastly, this work strongly suggests that dietary methionine supplementation for 4 weeks improves disease resistance against *Phdp* in a dose-dependent manner.

## Author contributions

MM, LC, and BC conceived the experiments. MM and FF conducted the experimental trial. RA and SF-B assisted with analytical procedures. MM directed most laboratory techniques and wrote the manuscript under the supervision of RA, LC, and BC. JD formulated and produced the experimental diets. All authors contributed to and approved the manuscript.

### Conflict of interest statement

The authors declare that the research was conducted in the absence of any commercial or financial relationships that could be construed as a potential conflict of interest.
