# Supplementary material for: Dietary Methionine Improves the European Seabass (Dicentrarchus labrax) Immune Status, Inflammatory Response, and Disease Resistance
Source: Front Immunol. 2018 Nov 20;9:2672. doi: 10.3389/fimmu.2018.02672 (PMC6256742; doi:10.3389/fimmu.2018.02672)
Supplement: Supplementary file 1 [file Table_1.docx]

Marina Machado^1,2,3,4*^, Rita Azeredo^1,3^, Filipa Fontinha^1,3^, Sergio Fernandez-Boo^1^, Luis E.C. Conceição^5^, Jorge Dias^5^ and Benjamín Costas^1,3*^

^1^ Centro Interdisciplinar de Investigação Marinha e Ambiental (CIIMAR), Terminal de Cruzeiros do Porto de Leixões, Av. General Norton de Matos s/n, 4450-208 Matosinhos
Portugal.

^2^ Instituto de Investigação e Inovação em Saúde (i3S), Universidade do Porto, Rua Alfredo Allen, 208, 4200-135 Porto, Portugal.

^3^ Instituto de Ciências Biomédicas Abel Salazar (ICBAS-UP), Universidade do Porto, Rua de Jorge Viterbo Ferreira nº 228, 4050-313 Porto, Portugal.

^4^ Instituto de Biologia Molecular e Celular, Universidade do Porto, Rua Alfredo Allen, 208, 4200-135 Porto, Portugal.

^5^ Sparos Lda, Area Empresarial de Marim, Lote C, Olhão, Portugal.

***Corresponding authors:**

Marina Machado

mcasimiro@ciimar.up.pt

Benjamín Costas

bcostas@ciimar.up.pt

**Supplementary File**

**Table S1.** Forward and reverse primers for real-time PCR.

| Acronym | Gene Bank ID | Eff^1^ | AT^2^ | Product lenght^3^ | Forward primer sequence | Reverse primer sequence |
| --- | --- | --- | --- | --- | --- | --- |
| *40s* | HE978789.1 | 92.96 | 55 | 79 | TGATTGTGACAGACCCTCGTG | CACAGAGCAATGGTGGGGAT |
| *il1β* | AJ311925 | 96.70 | 57 | 105 | AGCGACATGGTGCGATTTCT | CTCCTCTGCTGTGCTGATGT |
| *il8* | AM490063.1 | 102.87 | 55 | 140 | CGCTGCATCCAAACAGAGAGCAAAC | TCGGGGTCCAGGCAAACCTCTT |
| *il6* | AM490062.1 | 134.62 | 55 | 81 | AGGCACAGAGAACACGTCAAA | AAAAGGGTCAGGGCTGTCG |
| *tgfβ* | AM421619.1 | 105.56 | 55 | 143 | ACCTACATCTGGAACGCTGA | TGTTGCCTGCCCACATAGTAG |
| *tnfα* | DQ070246.1 | 108.81 | 55 | 112 | AGCCACAGGATCTGGAGCTA | GTCCGCTTCTGTAGCTGTCC |
| *cox 2* | AJ630649.1 | 81.30 | 61 | 160 | CATTCTTTGCCCAGCACTTCACC | AGCTTGCCATCCTTGAAGAGTC |
| *il10* | [AM268529.1](https://www.ncbi.nlm.nih.gov/entrez/viewer.fcgi?db=nucleotide&id=148472689) | 116.00 | 55 | 164 | ACCCCGTTCGCTTGCCA | CATCTGGTGACATCACTC |
| *ccr3* | DLAgn_00000190 | 117.69 | 55 | 381 | GCACTGTATGTGACCCGGAA | AGCAGATGTTTTGTTATCAGGACT |
| *cxcr4* | FN687464.1 | 93.43 | 57 | 171 | ACC AGA CCT TGT GTT TGC CA | ATG AAG CCC ACC AGG ATG TG |
| *sod* | CX660893.1 | 103.03 | 55 | 71 | GGAGAGTGATTCAGCCCCTG | GGAAACCATGCTCACCAGGA |
| *gpx* | DT044993 | 94.17 | 57 | 176 | GTTTGGACATCAGGAGAACTGC | CATCGCTGGGGTATGGAAGC |
| *hep* | DQ131605.1 | 94.17 | 57 | 152 | CTGGAGGAGCCAATGAGCAA | TGGAGAGAGCATCAGAGCAC |
| *noxin* | KM225775 | 106.97 | 62 | 72 | AGAGGTTGGTGGAGAACTTGGATGGA | CGACAGCCTTCATCAACAATGTGGATCT |
| *mhc II* | AM113468.1 | 98.15 | 55 | 81 | ATCCCTCCATGTTGGTCTGC | CTTCCTGTCCGTCTCTGAGC |
| *c3zeta* | DLAgn_00052540 | 131.01 | 55 | 819 | GCCACCAAAGACACCTACGA | GTGTTGAACGCAGGAGGGTA |
| *cd8 β* | DLAgn_00090370 | 113.81 | 55 | 651 | CGGAACCCAAAAGGCCAAAG | TAGGCTGTAGATGCAGTGCT |
| *tlr 9* | KX399289 | 115.17 | 55 | 100 | TCTTGGTTTGCCGACTTCTTGCGT | TACTGTTGCCCTGTTGGGACTCTGG |
| *tlr 2* | DLAgn_00214290 | 118.84 | 55 | 615 | GGCTCCACCACCTACCTAGA | AGGTGGATCTTCTGTCAAAAATGG |
| *mcsf1r1* | DLAgn_00109630 | 125.93 | 55 | 807 | TTGACCGTGGAGAAGGCAAA | AGAATGGACCTCAGCCAGTC |
| *mmp 9* | FN908863.1 | 98.44 | 57 | 166 | TGT GCC ACC ACA GAC AAC TT | TTC CAT CTC CAC GTC CCT CA |
| *c3* | HM563078.1 | 111.48 | 57 | 165 | CAGTGGGAATCTGTGGGCTT | GGCAAACACCTTGGCAAC |
| *mtor* | DLAgn_00134190 | 127.25 | 55 | 848 | CAGAACCAAGGACGTGACGA | TGGTAGTAGAGGTCCCAGGC |
| *casp 3* | DQ345773.1 | 130.10 | 55 | 235 | CTGATTTGGATCCAGGCATT | CGGTCGTAGTGTTCCTCCAT |
| *casp 1* | DQ198377.1 | 124.32 | 55 | 190 | GTGTTTCAGATGCGGGAGGA | ATTTAAGTTAACTCACCGGGGG |
| *stat 3* | DLAgn_00192560 | 110.68 | 55 | 275 | GACATCAGCGGAAAGACCCA | GGGGTGACGCAGATGAACTT |
| *mc2r* | FR870225.1 | 108.68 | 55 | 676 | GGAACAGGAACCTCCACTCG | ACCACGTGTAGCTGGAACAG |
| *hsp 70* | AY423555.2 | 134.14 | 55 | 88 | ACAAAGCAGACCCAGACCTTCACCA | TGGTCATAGCACGTTCGCCCTCA |
| *hsp 90* | AY395632.1 | 105.63 | 55 | 112 | GCTGACAAGAACGACAAGGCTGTGA | AGATGCGGTTGGAGTGGGTCTGT |
| *sat 1* | KM225772 | 97.55 | 63 | 55 | GCATCATCGCTGAAATCCAAGGAGAGAACA | CCAACCACCTTCAGGCCGTCACT |
| *amd 1* | KM225770 | 118.64 | 57.2 | 63 | CTGACGGAACTTACTGGACCATC | CGAAGCTGACGTAGGAGAACTC |

^1^ Efficiency of PCR reactions were calculated from serial dilutions of tissue RT reactions in the validation procedure.

^2^ Annealing temperature (°C)

^3^ Amplicon (nt)

**Table S2.** Quantitative expression of immune-related genes in the head-kidney of European seabass fed the dietary treatments during 2 and 4 weeks.

| Parameters | |  | Dietary treatments | | | | | | | |
| --- | --- | --- | --- | --- | --- | --- | --- | --- | --- | --- |
|  |  |  | CTRL | |  | MET 0.5 | |  | MET 1 | |
|  |  |  | 2 weeks | 4 weeks |  | 2 weeks | 4 weeks |  | 2 weeks | 4 weeks |
| *il1β* | Normalized mRNA expression |  | 0.114 ± 0.090 | 0.021 ± 0.029 |  | 0.054±0.043 | 0.028 ± 0.041 |  | 0.020 ± 0.020 | 0.028 ± 0.041 |
| *il8* |  |  | 0.094 ± 0.103 | 0.048 ± 0.086 |  | 0.020 ± 0.028 | 0.046 ± 0.060 |  | 0.029 ± 0.038 | 0.051 ± 0.074 |
| *il6* |  |  | 0.008 ± 0.008 | 0.003 ± 0.004 |  | 0.004 ± 0.004 | 0.003 ± 0.006 |  | 0.001 ± 0.002 | 0.003 ± 0.005 |
| *tgfβ* |  |  | 0.003 ± 0.001 | 0.003 ± 0.001 |  | 0.004 ± 0.002 | 0.003 ± 0.001 |  | 0.003 ± 0.002 | 0.004 ± 0.003 |
| *tnfα* |  |  | 0.079 ± 0.084 | 0.079 ± 0.114 |  | 0.031 ± 0.030 | 0.098 ± 0.215 |  | 0.017 ± 0.018 | 0.033 ± 0.051 |
| *cox 2* |  |  | 0.055 ± 0.058 | 0.134 ± 0.178 |  | 0.071 ± 0.080 | 0.243 ± 0.415 |  | 0.024 ± 0.025 | 0.065 ± 0.094 |
| *il10* |  |  | 0.051 ± 0.059 | 0.017 ± 0.028 |  | 0.020 ± 0.018 | 0.010 ± 0.014 |  | 0.011 ± 0.014 | 0.008 ± 0.011 |
| *ccr3* |  |  | 0.008 ± 0.008 | 0.021 ± 0.038 |  | 0.005 ± 0.005 | 0.005 ± 0.007 |  | 0.003 ± 0.003 | 0.004 ± 0.008 |
| *cxcr4* |  |  | 0.121 ± 0.197 | 0.016 ± 0.023 |  | 0.101 ± 0.185 | 0.061 ± 0.112 |  | 0.052 ± 0.074 | 0.150 ± 0.302 |
| *sod* |  |  | 0.157 ± 0.176 | 0.194 ± 0.249 |  | 0.129 ± 0.090 | 0.095 ± 0.065 |  | 0.054 ± 0.015 | 0.074 ± 0.070 |
| *gpx* |  |  | 0.276 ± 0.147 | 0.405 ± 0.250 |  | 0.288 ± 0.099 | 0.212 ± 0.059 |  | 0.230 ± 0.093 | 0.244 ± 0.096 |
| *hep* |  |  | 0.074 ± 0.377 | 0.726 ± 1.128 |  | 0.523 ± 0.888 | 0.591 ± 0.995 |  | 0.080 ± 0.086 | 1.612 ± 3.026 |
| *noxin* |  |  | 0.024 ± 0.025 | 0.023 ± 0.037 |  | 0.009 ± 0.008 | 0.012 ± 0.013 |  | 0.003 ± 0.005 | 0.008 ± 0.011 |
| *mhc II* |  |  | 0.688 ± 0.508 | 0.410 ± 0.354 |  | 0.469 ± 0.489 | 1.205 ± 1.304 |  | 0.220 ± 0.177 | 0.709 ± 0.509 |
| *c3zeta* |  |  | 0.000 ± 0.000 | 0.021 ± 0.037 |  | 0.005 ± 0.005 | 0.005 ± 0.007 |  | 0.003 ± 0.003 | 0.004 ± 0.008 |
| *cd8 β* |  |  | 0.032 ± 0.028 | 0.093 ± 0.132 |  | 0.013 ± 0.019 | 0.018 ± 0.029 |  | 0.010 ± 0.012 | 0.016 ± 0.024 |
| *tlr 9* |  |  | 0.020 ± 0.021 | 0.054 ± 0.074 |  | 0.011 ± 0.011 | 0.015 ± 0.021 |  | 0.007 ± 0.008 | 0.012 ± 0.018 |
| *tlr 2* |  |  | 0.005 ± 0.005 | 0.007 ± 0.016 |  | 0.002 ± 0.001 | 0.003 ± 0.005 |  | 0.003 ± 0.002 | 0.003 ± 0.005 |
| *mcsf1r1* |  |  | 0.004 ± 0.004 | 0.020 ± 0.031 |  | 0.002 ± 0.002 | 0.002 ± 0.003 |  | 0.001 ± 0.001 | 0.003 ± 0.004 |
| *mmp 9* |  |  | 0.095 ± 0.087 | 0.057 ± 0.065 |  | 0.055 ± 0.040 | 0.040 ± 0.029 |  | 0.045 ± 0.031 | 0.061 ± 0.074 |
| *c3* |  |  | 0.091 ± 0.131 | 0.055 ± 0.123 |  | 0.084 ± 0.106 | 0.044 ± 0.077 |  | 0.010 ± 0.013 | 0.096 ± 0.142 |
| *mtor* |  |  | 0.001 ± 0.001 | 0.001 ± 0.001 |  | 0.000 ± 0.001 | 0.000 ± 0.001 |  | 0.000 ± 0.000 | 0.001 ± 0.002 |
| *casp 3* |  |  | 0.065 ± 0.079 | 0.208 ± 0.304 |  | 0.081 ± 0.129 | 0.021 ± 0.024 |  | 0.027 ± 0.038 | 0.019 ± 0.030 |
| *casp 1* |  |  | 0.039 ± 0.046 | 0.025 ± 0.036 |  | 0.031 ± 0.050 | 0.006 ± 0.009 |  | 0.006 ± 0.011 | 0.019 ± 0.030 |
| *stat 3* |  |  | 0.405 ± 0.393 | 0.488 ± 0.693 |  | 0.410 ± 0.503 | 0.584 ± 0.737 |  | 0.150 ± 0.145 | 0.199 ± 0.250 |
| *mc2r* |  |  | 0.004 ± 0.003^a^ | 0.002 ± 0.002 |  | 0.001 ± 0.001^b^ | 0.003 ± 0.003 |  | 0.001 ± 0.001^b^ | 0.001 ± 0.001 |
| *hsp 70* |  |  | 0.026 ± 0.019 | 0.045 ± 0.069 |  | 0.014 ± 0.010 | 0.011 ± 0.008 |  | 0.013 ± 0.009 | 0.014 ± 0.017 |
| *hsp 90* |  |  | 0.872 ± 0.628 | 1.190 ± 1.661 |  | 0.533 ± 0.375 | 0.424 ± 0.357 |  | 0.373 ± 0.211 | 0.478 ± 0.555 |
| *sat 1* |  |  | 0.044 ± 0.019 | 0.025 ± 0.013 |  | 0.030 ± 0.012 | 0.021±0.009 |  | 0.023 ± 0.009 | 0.021 ± 0.011 |
| *amd 1* |  |  | 0.006 ± 0.011 | 0.004 ±0.007 |  | 0.007 ± 0.006 | 0.002±0.002 |  | 0.002 ± 0.002 | 0.002 ± 0.004 |

| Two-way ANOVA | | | | | | | |  |
| --- | --- | --- | --- | --- | --- | --- | --- | --- |
|  |  |  |  |  |  | Diet | | |
| Parameters |  | Time | Diet | Time × Diet |  | CTRL | MET 0.5 | MET 1 |
| *il1β* |  | - | 0.028 | - |  | A | AB | B |
| *il8* |  | - | - | - |  | - | - | - |
| *il6* |  | - | - | - |  | - | - | - |
| *tgfβ* |  | - | - | - |  | - | - | - |
| *tnfα* |  | - | - | - |  | - | - | - |
| *cox 2* |  | - | - | - |  | - | - | - |
| *il10* |  | - | - | - |  | - | - | - |
| *ccr3* |  | - | - | - |  | - | - | - |
| *cxcr4* |  | - | - | - |  | - | - | - |
| *sod* |  | - | - | - |  | - | - | - |
| *gpx* |  | - | - | - |  | - | - | - |
| *hep* |  | - | - | - |  | - | - | - |
| *noxin* |  | - | 0.047 | - |  | A | AB | B |
| *mhc II* |  | - | - | - |  | - | - | - |
| *c3zeta* |  | - | - | - |  | - | - | - |
| *cd8 β* |  | - | 0.038 | - |  | A | B | B |
| *tlr 9* |  | - | - | - |  | - | - | - |
| *tlr 2* |  | - | - | - |  | - | - | - |
| *mcsf1r1* |  | - | - | - |  | - | - | - |
| *mmp 9* |  | - | - | - |  | - | - | - |
| *c3* |  | - | - | - |  | - | - | - |
| *mtor* |  | - | - | - |  | - | - | - |
| *casp 3* |  | - | 0.039 | - |  | A | AB | B |
| *casp 1* |  | - | - | - |  | - | - | - |
| *stat 3* |  | - | - | - |  | - | - | - |
| *mc2r* |  | - | - | 0.019 |  | - | - | - |
| *hsp 70* |  | - | - | - |  | - | - | - |
| *hsp 90* |  | - | - | - |  | - | - | - |
| *sat 1* |  | 0.008 | 0.020 | - |  | A | AB | B |
| *amd 1* |  | - | - | - |  | - | - | - |

Values are presented as means ± SD (n=9). P-values from two-way ANOVA (p ≤0.05). If interaction was significant, Tukey post hoc test was used to identify differences in the experimental treatments. Different lowercase letters stand for significant differences among dietary treatments for the same time while different symbols stands for significant differences between times for the same diet. Different capital letters indicate differences among times regardless diets and among diets regardless time.

**Table S3.** Quantitative expression of immune-related genes in the head-kidney of European seabass fed the dietary treatments at 4 weeks (0 h), 4, 24 and 48 h after infection

| Parameters | |  | Dietary treatments | | | | | | | | | | | | | |
| --- | --- | --- | --- | --- | --- | --- | --- | --- | --- | --- | --- | --- | --- | --- | --- | --- |
|  |  |  | CTRL | | | |  | MET 0.5 | | | |  | MET 1 | | | |
|  |  |  | 0h | 4h | 24h | 48h |  | 0h | 4h | 24h | 48h |  | 0h | 4h | 24h | 48h |
| *il1β* | Normalized mRNA expression |  | 0.021 ± 0.029 | 0.178 ± 0.160 | 0.243 ± 0.107 | 0.219 ± 0.088 |  | 0.028 ± 0.041 | 0.085 ± 0.077 | 0.222 ± 0.124 | 0.118 ± 0.100 |  | 0.028 ± 0.041 | 0.236 ± 0.284 | 0.186 ± 0.154 | 0.133 ± 0.122 |
| *il8* |  |  | 0.048 ± 0.086 | 0.430 ± 0.651 | 0.199 ± 0.087 | 0.254 ± 0.099 |  | 0.046 ± 0.060 | 0.128 ± 0.070 | 0.352 ± 0.243 | 0.079 ± 0.030 |  | 0.051 ± 0.074 | 0.073 ± 0.046 | 0.614 ± 0.738 | 0.226 ± 0.267 |
| *il6* |  |  | 0.003 ± 0.004 | 0.000 ± 0.000 | 0.015 ± 0.007 | 0.007 ± 0.005 |  | 0.003 ± 0.006 | 0.012 ± 0.009 | 0.009 ± 0.009 | 0.001 ± 0.001 |  | 0.003 ± 0.005 | 0.004 ± 0.006 | 0.021 ± 0.026 | 0.002 ± 0.003 |
| *tgfβ* |  |  | 0.003 ± 0.001 | 0.002 ± 0.001 | 0.001 ± 0.000 | 0.004 ± 0.002^b^ |  | 0.003 ± 0.001 | 0.003 ± 0.001 | 0.002 ± 0.002 | 0.003 ± 0.007^b^ |  | 0.004 ± 0.003* | 0.003 ± 0.001* | 0.007 ± 0.008^*£^ | 0.013 ± 0.010^a£^ |
| *tnfα* |  |  | 0.079 ± 0.114 | 0.129 ± 0.099 | 0.156 ± 0.129 | 0.186 ± 0.124 |  | 0.098 ± 0.215 | 0.125 ± 0.089 | 0.131 ± 0.082 | 0.023 ± 0.009 |  | 0.033 ± 0.051* | 0.017 ± 0.030* | 0.355 ± 0.225^£^ | 0.093 ± 0.097^£^* |
| *cox 2* |  |  | 0.134 ± 0.178 | 0.732 ± 0.439 | 0.974 ± 0.537 | 0.567 ± 0.703 |  | 0.243 ± 0.415 | 0.632 ± 0.370 | 0.842 ± 0.649 | 0.034 ± 0.025 |  | 0.065 ± 0.094 | 0.237 ± 0.250 | 1.324 ± 0.766 | 0.146 ± 0.116 |
| *il10* |  |  | 0.017 ± 0.028 | 0.097 ± 0.072 | 0.050 ± 0.058 | 0.099 ± 0.078 |  | 0.010 ± 0.014 | 0.010 ± 0.014 | 0.080 ± 0.067 | 0.100 ± 0.103 |  | 0.008 ± 0.011 | 0.021 ± 0.019 | 0.060 ± 0.076 | 0.135 ± 0.136 |
| *ccr3* |  |  | 0.021 ± 0.038 | 0.036 ± 0.033 | 0.036 ± 0.026^b^ | 0.049 ± 0.038 |  | 0.005 ± 0.007 | 0.032 ± 0.025 | 0.054 ± 0.043^ab^ | 0.013 ± 0.009 |  | 0.004 ± 0.008* | 0.002 ± 0.003* | 0.155 ± 0.163^a£^ | 0.025 ± 0.030* |
| *cxcr4* |  |  | 0.016 ± 0.023 | 0.470 ± 0.541 | 3.520 ± 2.830 | 2.479 ± 1.912 |  | 0.061 ± 0.112 | 0.137 ± 0.100 | 4.217 ± 3.044 | 0.382 ± 0.229 |  | 0.150 ± 0.302 | 0.038 ± 0.023 | 4.123 ± 2.974 | 0.763 ± 0.926 |
| *hep* |  |  | 0.726 ± 1.128 | 1.600 ± 2.825 | 2.809 ± 1.124 | 2.599 ± 1.118 |  | 0.591 ± 0.995 | 3.117 ± 2.568 | 3.476 ± 2.873 | 0.385 ± 0.297 |  | 1.612 ± 3.026 | 0.822 ± 0.479 | 3.099 ± 1.751 | 2.321 ± 2.198 |
| *noxin* |  |  | 0.023 ± 0.037 | 0.034 ± 0.022 | 0.069 ± 0.045 | 0.070 ± 0.025 |  | 0.012 ± 0.013 | 0.038 ± 0.028 | 0.060 ± 0.037 | 0.014 ± 0.010 |  | 0.008 ± 0.011 | 0.012 ± 0.013 | 0.069 ± 0.038 | 0.034 ± 0.029 |
| *mhc II* |  |  | 0.410 ± 0.354 | 0.231 ± 0.481 | 2.192 ± 1.276 | 2.847 ± 1.425 |  | 1.205 ± 1.304 | 1.545 ± 1.397 | 2.224 ± 1.328 | 1.014 ± 0.935 |  | 0.709 ± 0.509 | 0.352 ± 0.349 | 2.310 ± 1.560 | 1.636 ± 1.521 |
| *tlr9* |  |  | 0.054 ± 0.074 | 0.071 ± 0.057 | 0.098 ± 0.099 | 0.119 ± 0.080 |  | 0.015 ± 0.021 | 0.061 ± 0.036 | 0.114 ± 0.084 | 0.016 ± 0.011 |  | 0.012 ± 0.018 | 0.014 ± 0.020 | 0.146 ± 0.111 | 0.059 ± 0.064 |
| *tlr2* |  |  | 0.007 ± 0.016 | 0.032 ± 0.033 | 0.022 ± 0.012 | 0.000 ± 0.000 |  | 0.003 ± 0.005 | 0.015 ± 0.012 | 0.049 ± 0.058 | 0.002 ± 0.001 |  | 0.003 ± 0.005 | 0.004 ± 0.003 | 0.094 ± 0.131 | 0.001 ± 0.002 |
| *mcsf1r1* |  |  | 0.020 ± 0.031 | 0.022 ± 0.023 | 0.013 ± 0.012 | 0.019 ± 0.019 |  | 0.002 ± 0.003 | 0.010 ± 0.009 | 0.017 ± 0.015 | 0.016 ± 0.011 |  | 0.003 ± 0.004 | 0.002 ± 0.002 | 0.036 ± 0.031 | 0.008 ± 0.009 |
| *mmp9* |  |  | 0.057 ± 0.065 | 1.498 ± 2.087 | 0.354 ± 0.155 | 0.434 ± 0.377 |  | 0.040 ± 0.029 | 0.271 ± 0.208 | 0.401 ± 0.245 | 0.179 ± 0.116 |  | 0.061 ± 0.074 | 0.613 ± 0.790 | 0.401 ± 0.245 | 0.187 ± 0.151 |
| *c3* |  |  | 0.055 ± 0.123 | 0.389 ± 0.385 | 0.286 ± 0.137 | 0.319 ± 0.216 |  | 0.044 ± 0.077 | 0.236 ± 0.121 | 0.329 ± 0.187 | 0.293 ± 0.283 |  | 0.096 ± 0.142 | 0.049 ± 0.054 | 0.350 ± 0.192 | 0.174 ± 0.188 |
| *mtor* |  |  | 0.001 ± 0.001 | 0.007 ± 0.006 | 0.005 ± 0.002 | 0.005 ± 0.003 |  | 0.000 ± 0.001 | 0.003 ± 0.002 | 0.005 ± 0.004 | 0.001 ± 0.001 |  | 0.001 ± 0.002 | 0.001 ± 0.001 | 0.004 ± 0.002 | 0.001 ± 0.000 |
| *casp 3* |  |  | 0.208 ± 0.304 | 0.331 ± 0.191 | 0.200 ± 0.087 | 0.296 ± 0.175 |  | 0.021 ± 0.024 | 0.134 ± 0.095 | 0.337 ± 0.272 | 0.092 ± 0.083 |  | 0.019 ± 0.030 | 0.048 ± 0.053 | 0.175 ± 0.111 | 0.148 ± 0.166 |
| *casp 1* |  |  | 0.025 ± 0.036 | 0.061 ± 0.051 | 0.041 ± 0.024 | 0.074 ± 0.039 |  | 0.006 ± 0.009 | 0.037 ± 0.028 | 0.053 ± 0.041 | 0.019 ± 0.015 |  | 0.019 ± 0.030 | 0.010 ± 0.011 | 0.075 ± 0.062 | 0.031 ± 0.031 |
| *stat 3* |  |  | 0.488 ± 0.693 | 1.466 ± 0.987 | 1.147 ± 0.547 | 1.863 ± 0.965 |  | 0.584 ± 0.737 | 0.959 ± 0.659 | 1.780 ± 1.190 | 0.732 ± 0.586 |  | 0.199 ± 0.250 | 0.554 ± 0.240 | 2.121 ± 1.584 | 1.145 ± 1.116 |
| *m2cr* |  |  | 0.002 ± 0.002 | 0.002 ± 0.001 | 0.024 ± 0.029 | 0.011± 0.009 |  | 0.003 ± 0.003 | 0.006 ± 0.006 | 0.000 ± 0.000 | 0.019 ± 0.016 |  | 0.001 ± 0.001 | 0.006 ± 0.0010 | 0.012 ± 0.018 | 0.024 ± 0.041 |
| *hsp70* |  |  | 0.045 ± 0.069 | 0.061 ± 0.057 | 0.054 ± 0.041 | 0.052 ± 0.038 |  | 0.011 ± 0.008 | 0.035 ± 0.030 | 0.054 ± 0.035 | 0.007 ± 0.003 |  | 0.014 ± 0.017 | 0.010 ± 0.009 | 0.127 ± 0.122 | 0.034 ± 0.038 |
| *hsp90* |  |  | 1.190 ± 1.661 | 1.656 ± 1.327 | 2.007 ± 1.612 | 2.648 ± 2.219 |  | 0.424 ± 0.357 | 1.225 ± 0.793 | 2.360 ± 1.803 | 0.346 ± 0.159 |  | 0.478 ± 0.555 | 0.296 ± 0.293 | 3.999 ± 3.368 | 1.099 ± 1.020 |
| *sat 1* |  |  | 0.025 ± 0.013 | 0.004 ± 0.002 | 0.023 ± 0.012 | 0.023 ± 0.026^ab^ |  | 0.021±0.009 | 0.021 ± 0.013 | 0.034 ± 0.029 | 0.004 ± 0.003^b^ |  | 0.021 ± 0.011 | 0.023 ± 0.018 | 0.034 ± 0.015 | 0.040 ± 0.020^a^ |
| *amd 1* |  |  | 0.004 ±0.007 | 0.113 ± 0.064^b^ | 0.054 ± 0.012 | 0.216 ± 0.196 |  | 0.002±0.002* | 0.417 ± 0.327^ab£^ | 0.031 ± 0.017^£^ | 0.046 ± 0.040^£^ |  | 0.002 ± 0.004 | 0.753 ± 0.468^a£^ | 0.081 ± 0.046 | 0.256 ± 0.180 |

| Two-way ANOVA | | | | | | | | | | | |  |  |
| --- | --- | --- | --- | --- | --- | --- | --- | --- | --- | --- | --- | --- | --- |
|  |  |  |  |  |  | Time | | | |  | Diet | | |
| Parameters |  | Time | Diet | Time × Diet |  | 0h | 4h | 24h | 48h |  | CTRL | MET 0.5 | MET 1 |
| *il1β* |  | 0.003 | - | - |  | B | A | A | AB |  | - | - | - |
| *il8* |  | 0.001 | - | - |  | B | A | AB | AB |  | - | - | - |
| *il6* |  | < 0.001 | - | - |  | B | B | A | B |  | - | - | - |
| *tgfβ* |  | 0.012 | < 0.001 | 0.195 |  | B | B | AB | A |  | B | B | A |
| *tnfα* |  | 0.033 | - | 0.032 |  | B | AB | A | AB |  | - | - | - |
| *cox 2* |  | < 0.001 | - | - |  | C | B | A | CB |  | - | - | - |
| *il10* |  | 0.013 | - | - |  | B | AB | AB | A |  | - | - | - |
| *ccr3* |  | 0.001 | - | 0.021 |  | B | B | A | AB |  | - | - | - |
| *cxcr4* |  | < 0.001 | - | - |  | BC | C | A | B |  | - | - | - |
| *hep* |  | 0.019 | - | - |  | B | AB | A | AB |  | - | - | - |
| *noxin* |  | < 0.001 | - | - |  | B | BC | A | C |  | - | - | - |
| *mhc II* |  | < 0.001 | - | - |  | B | B | A | A |  | - | - | - |
| *tlr9* |  | < 0.001 | - | - |  | B | B | A | AB |  | - | - | - |
| *tlr2* |  | 0.008 | - | - |  | B | AB | A | B |  | - | - | - |
| *mcsf1r1* |  | - | - | - |  | - | - | - | - |  | - | - | - |
| *mmp9* |  | 0.019 | - | - |  | B | A | AB | AB |  | - | - | - |
| *c3* |  | 0.002 | - | - |  | B | AB | A | A |  | - | - | - |
| *mtor* |  | < 0.001 | 0.005 | - |  | B | A | A | AB |  | A | AB | B |
| *casp 3* |  | - | 0.024 | - |  | - | - | - | - |  | A | AB | B |
| *casp 1* |  | 0.008 | - | - |  | B | AB | A | AB |  | - | - | - |
| *stat 3* |  | < 0.001 | - | - |  | B | AB | A | A |  | - | - | - |
| *m2cr* |  | 0.014 | - | - |  | B | AB | AB | A |  | - | - | - |
| *hsp70* |  | 0.012 | - | - |  | B | AB | A | AB |  | - | - | - |
| *hsp90* |  | 0.001 | - | - |  | B | B | A | AB |  | - | - | - |
| *sat 1* |  | - | - | 0.041 |  | - | - | - | - |  | - | - | - |
| *amd 1* |  | < 0.001 | 0.006 | 0.003 |  | C | A | BC | B |  | B | AB | A |

Values are presented as means ± SD (n=6). P-values from two-way ANOVA (p ≤0.05). If interaction was significant, Tukey post hoc test was used to identify differences in the experimental treatments. Different lowercase letters stand for significant differences among dietary treatments for the same time while different symbols stands for significant differences between times for the same diet. Different capital letters indicate differences among times regardless diets and among diets regardless time.
